# Genome-resolved metagenomics reveals role of iron metabolism in drought-induced rhizosphere microbiome dynamics

**DOI:** 10.1038/s41467-021-23553-7

**Published:** 2021-05-28

**Authors:** Ling Xu, Zhaobin Dong, Dawn Chiniquy, Grady Pierroz, Siwen Deng, Cheng Gao, Spencer Diamond, Tuesday Simmons, Heidi M.-L. Wipf, Daniel Caddell, Nelle Varoquaux, Mary A. Madera, Robert Hutmacher, Adam Deutschbauer, Jeffery A. Dahlberg, Mary Lou Guerinot, Elizabeth Purdom, Jillian F. Banfield, John W. Taylor, Peggy G. Lemaux, Devin Coleman-Derr

**Affiliations:** 1 Department of Plant and Microbial Biology, University of California, Berkeley, CA USA; 2 State Key Laboratory of Plant Physiology and Biochemistry, Department of Microbiology and Immunology, College of Biological Sciences, China Agricultural University, Beijing, China; 3 Department of Energy, Environmental Genomics and Systems Biology Division, Lawrence Berkeley National Laboratory, Berkeley, CA USA; 4 Department of Earth and Planetary Science, University of California, Berkeley, CA USA; 5 Plant Gene Expression Center, USDA-ARS, Albany, CA USA; 6 CNRS, University Grenoble Alpes, TIMC-IMAG, Grenoble, France; 7 Westside Research & Extension Center, UC Department of Plant Sciences, University of California, Davis, CA USA; 8 Kearney Agricultural Research & Extension Center, Parlier, CA USA; 9 Department of Biological Scienes, Dartmouth College, Hanover, NH USA; 10 Department of Statistics, University of California, Berkeley, CA USA

**Keywords:** Soil microbiology, Metagenomics, Microbe, Plant molecular biology, Drought

## Abstract

Recent studies have demonstrated that drought leads to dramatic, highly conserved shifts in the root microbiome. At present, the molecular mechanisms underlying these responses remain largely uncharacterized. Here we employ genome-resolved metagenomics and comparative genomics to demonstrate that carbohydrate and secondary metabolite transport functionalities are overrepresented within drought-enriched taxa. These data also reveal that bacterial iron transport and metabolism functionality is highly correlated with drought enrichment. Using time-series root RNA-Seq data, we demonstrate that iron homeostasis within the root is impacted by drought stress, and that loss of a plant phytosiderophore iron transporter impacts microbial community composition, leading to significant increases in the drought-enriched lineage, Actinobacteria. Finally, we show that exogenous application of iron disrupts the drought-induced enrichment of Actinobacteria, as well as their improvement in host phenotype during drought stress. Collectively, our findings implicate iron metabolism in the root microbiome’s response to drought and may inform efforts to improve plant drought tolerance to increase food security.

## Abstract (web summary)

Advances in omics provide a tool to understand mechanisms for plant–microbial interactions under stress. Here the authors apply genome-resolved metagenomics to investigate sorghum and its microbiome responses to drought, identifying an unexpected role of iron metabolism.

## Introduction

Organisms living in terrestrial ecosystems must be able to adapt to dry conditions that result from shifts in water availability due to diurnal and seasonal fluctuations^1–3^. Plants, as sessile organisms, have adapted strategies to tolerate, resist, or avoid the stress imposed by drying environments. To respond to moisture gradients in soil, plants alter their physiology, modify root growth and architecture, and close stomata on their aboveground segments^4^. Recently, it has been demonstrated that interactions with microbial partners above and below ground play a role in augmenting a plant’s ability to survive under drought^5–8^ and that drought significantly influences these interactions, altering both structure and function of the root microbiome^9–11^. As it is hypothesized that droughts in the future are likely to be more frequent, severe, and long-lasting than they have been in recent decades^12^; new and rapidly deployable solutions for improving drought tolerance in crops are needed. An increased understanding of the complex feedback between plants and microbes during and after drought will pave the way for harnessing the rhizosphere microbiome to increase the resilience of crop production to drought^13^.

A series of studies in diverse plant species^6,9–11,14,15^ has revealed that during drought, the plant root microbiome shifts in favor of the Actinobacteria and other Gram-positive taxa, which supplant the majority of Gram-negative lineages that typically reside there. This enrichment is most prevalent within the root endosphere but is also observable in the surrounding root−soil interface, known as the rhizosphere^6,16^. Additionally, this enrichment has been shown to be proportional to the strength and duration of drought and rapidly disappears when water returns to the root system^6^. Several hypotheses regarding the underlying causes of this conserved pattern in microbiome development have recently been proposed^16^, based primarily on putative genetic properties and metabolic capabilities of the enriched taxonomic groups and collective activity of these drought-stressed communities as assayed by metatranscriptomics^6^. However, more molecular evidence and a detailed dissection of microbial traits associated with the drought enrichment phenomena has been hampered by a lack of genetic and functional information for individual members of the rhizosphere community, which would allow comparative analysis between traits of drought-enriched and drought-depleted taxa.

Recent advances in the field of genome-resolved metagenomics now allow for the binning of genomes for individual community members from complex environments, including soils, enabling comparative genomic analyses of microorganisms for which isolates and isolate sequences are not available^17,18^. This strategy has been employed to better understand the turnover of soil C−N compounds, as well as to characterize the diversity of secondary metabolite biosynthesis genes in soil bacterial communities^17,18^. In this study, we use genome-resolved metagenomics to further explore microbial traits associated with drought enrichment phenotypes in the sorghum rhizosphere. The resulting data corroborate previous results that identified carbohydrate and secondary metabolite transport and metabolism as pathways correlated with bacterial enrichment under drought. Additionally, these new data help identifies new bacterial genetic traits associated with enrichment under drought stress, including the functional category inorganic ion transport. Exchange of inorganic ions, such as iron, nitrate, and phosphate, are hallmarks of many well-characterized plant microbe symbioses^19^, and recent work from other labs has demonstrated links between nutrient status in plant roots and overall microbiome regulation^20^. To further explore the relationship between inorganic ion transport, drought stress, and root microbiome regulation, we use a combination of transcriptomic, genetic, and inoculation experiments. Collectively, this study demonstrates the utility of genome-resolved metagenomics coupled with downstream reductionist experiments for dissecting plant−microbe interactions in the root-associated microbiome and improves our understanding of the mechanisms involved in previously characterized shifts drought imposes on the plant microbiome.

## Results

### Experimental design

To explore the impact of drought stress on root microbiome development, we conducted a large-scale field experiment in which drought-stressed and watered control sorghum plants were sampled weekly across 17 time points (TPs) in California’s Central Valley during the summer of 2016 (Supplementary Fig. 1). The Central Valley represents one of the most productive agricultural regions in the world and one hard hit by recent and intense drought^21^. For all plots analyzed in this study, one of two irrigation treatments (drought or control) was applied. For the drought treatment, no water was applied between planting (TP0) and the onset of flowering (TP8). To allow for an analysis of the impact of rewatering on microbiome development, plants in the drought treatment received irrigation shortly after TP8 and for the duration of the experiment (TP9−TP17). For the control treatment, irrigation (see "Methods") was applied throughout the majority of the experiment (TP3−17); as is customary to allow for sorghum seedling establishment, no water was applied to either drought or control treatments during the first two weeks post planting (TP0−TP2).

As the field site chosen for this experiment is naturally dry during the summer season, we first sought to determine the level of drought stress achieved by the drought treatment as compared to the watered control by quantifying the percent depletion of available soil moisture (ASM), which is correlated with the amount of water available to the plant root system. We observed that the depletion in ASM was significantly higher during drought treatment than in the control, but recovered rapidly to levels observed in the watered treatment following rewatering at TP9 (Supplementary Fig. 2). To directly measure the effect of the drought treatments on plant performance, measurements of the Crop Water Stress Index (CSWI), which serves as an approximation for reductions in the levels of active leaf transpiration^22,23^, were taken at a subset of time points throughout the experiment (Supplementary Fig. 3). These data demonstrate that the drought treatment led to corresponding significant increases in plant stress.

### Shotgun metagenome data

In a previously published study, an analysis of 16S rRNA data generated from this experimental design demonstrated that drought stress leads to a dramatic shift in the bacterial root microbiome. This shift is primarily characterized by an increase in the relative abundance of Actinobacteria and other Gram-positive lineages^6^, which continues to grow until rewatering after TP8. In the study presented here, shotgun metagenomic sequencing was performed for both rhizosphere and bulk soil samples collected at a subset of six time points analyzed in the previous study (TPs 3, 4, 8, 9, 10, and 11). These time points span early drought stress (TP3, 4), the peak of a drought (TP8), and subsequent drought recovery (TP9, 10, and 11) (Supplementary Fig. 1), and the resulting metagenomic data represent approximately 609 gigabases (Gb) of raw shotgun metagenomic sequence.

**Fig. 1:**
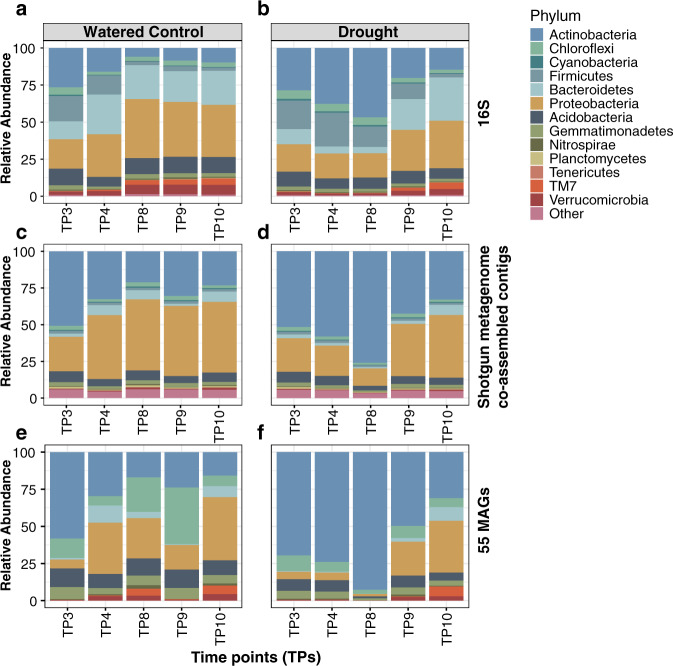
Relative abundance profiles of microbial taxa in the sorghum rhizosphere under drought. Percent relative abundance (*y*-axis) in the rhizosphere of the top 13 most abundant phyla for watered controls (**a**, **c**, **e**) and drought treatments (**b**, **d**, **f**) as measured by 16S rRNA gene amplicon sequencing (**a**, **b**), co-assembled contigs from shotgun metagenomic datasets (**c**, **d**), and all reads mapping to the 55 metagenome-assembled genomes (MAGs) (**e**, **f**). All five time points (TP3−TP10, shown on the *x*-axis) were selected based on the availability of data across all three data analysis types. All reads that mapped to other phyla or which were not classifiable at the phylum level are grouped into a fourteenth category, entitled Other. TP: time points.

To determine whether the shotgun metagenomics data reveal similar shifts in bacterial community composition following drought treatment as had been observed previously with amplicon analysis, we compared the taxonomic composition of our published 16S rRNA datasets^6^ with taxonomic profiles generated from co-assembled contigs generated from the shotgun dataset (Fig. 1a–d). As was observed in the 16S rRNA dataset (Fig. 1b), the shotgun data revealed a large increase in the relative abundance of Actinobacteria under drought stress in the rhizosphere community (Fig. 1d) and a weak enrichment in the surrounding soil under drought, as compared to control (Supplementary Fig. 4a–d). In addition, both datasets show that the enrichment of Actinobacteria under drought stress dissipates once the plants are rewatered, which occurred between TP8 and TP9 (Fig. 1b, d). Finally, the shotgun data corroborate a previously observed enrichment in Actinobacteria in the rhizosphere within the early stages of control-treated sorghum under control (Fig. 1c). However, in addition to the noted similarities in treatment-dependent longitudinal patterns in the amplicon and co-assembled contig data, several differences were also noted. For example, irrespective of treatment, the relative abundance represented by several phyla, including Bacteroidetes, Firmicutes, and Verrucomicrobia, is considerably smaller in the shotgun data than in the amplicon datasets, while the fraction ascribed to Actinobacteria is larger. Differential representation of these specific taxa within the databases used to ascribe taxonomy to 16S and shotgun datasets could explain these results; however, at the phylum level, representation for two of these lineages (Firmicutes and Bacteroidetes) is actually greater in the database used for shotgun data annotation (Supplementary Fig. 5a). Similar differences between 16S rRNA and shotgun data have been noted for these and other lineages in recent studies^24–26^, and could be due to differences in representation at the lower taxonomic level, differences in 16S rRNA copy number, or in PCR amplification bias in 16S rRNA datasets. We also noted that in the co-assembled contig dataset, a larger percentage of reads (approximately 10%) were not assignable to the 13 most abundant phyla observed in the 16S rRNA data; this is likely partially attributable to our ability to detect with shotgun data bacterial abundance from candidate phyla and other taxa not amplifiable by amplicon primers used in this study (Supplementary Fig. 5b). Collectively, these data demonstrate that while platform-specific differences can be observed, previously reported patterns of compositional shifts under drought are found in both amplicon and shotgun datasets.

### Drought-induced shifts in functional capacity in the rhizosphere microbiome

**Fig. 2:**
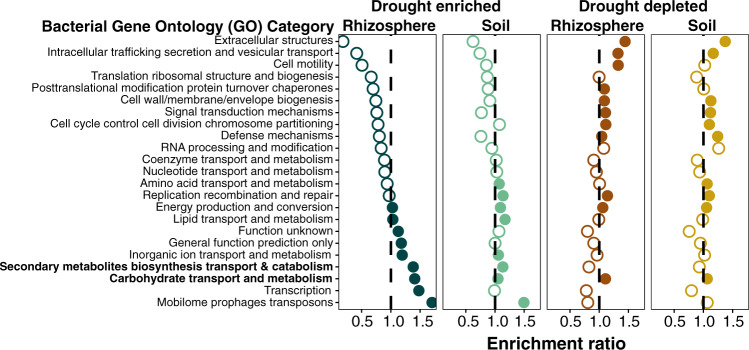
Bacterial gene functional capacity enrichment in rhizosphere and soil under drought. Bacteria gene ontology (GO) enrichment analysis for all genes derived from co-assembled contigs showing enrichment (panels one and two) or depletion (panels three and four) under drought for rhizospheres or soils at the peak of drought (TP8). The values on the *x*-axis indicate the fold enrichment ratio of the relative percentages of genes that are upregulated or downregulated under drought in each category relative to the total relative percentage of genes in the corresponding category within the entire dataset. Categories for which there were fewer than five differentially expressed genes were omitted. Solid circles indicate that the enrichment was significant (*p*-value of < = 0.05) in a one-sided hypergeometric test. Two categories discussed in more detail in the Results are shown in bold.

While amplicon data can allow for a broad survey of community composition across a large experimental design, shotgun data allow for a direct exploration of community functional capacity. In this study, we first used the shotgun datasets to identify microbial traits that are more prevalent within drought-stressed samples than control-treated samples. To achieve this, we predicted genes for all co-assembled contigs greater than 1,000 bp in length and assigned functional categories to all genes using the Clusters of Orthologous Groups (COG) categories, which resulted in 203,620 bacterial genes with COG annotations for downstream analysis. Previously published analysis of metatranscriptomic data from eight of these samples at peak of drought within control and drought treatments^6^ indicated an enrichment of gene activity belonging to carbohydrate transport and metabolism, secondary metabolite transport and metabolism, and amino acid transport and metabolism, within drought-treated rhizospheres, as compared to control. Here, the shotgun data also revealed enrichment of two of these categories under drought stress at the peak of drought, specifically carbohydrate transport and metabolism and secondary metabolite transport and metabolism (Fig. 2a). This suggests that the previously observed enrichment in these two functional activities is driven, at least in part, by a shift in overall functional capacity within the rhizosphere community in favor of these traits, and that on average, drought-enriched bacteria encode for a greater genetic repertoire for exchange or use of carbohydrates and secondary metabolites than those that are drought-depleted (Fig. 2a, c). Additionally, several other categories appear as enriched at peak of the drought in these co-assembled contig data, though they were not observed as enriched in prior metatranscriptome datasets; additional analyses at earlier and later time points reveal that the strength of enrichment for many of these categories builds as drought progresses, and wanes following rewatering after TP8 (Supplementary Fig. 6), mirroring the observed shifts in Actinobacteria across this time frame.

Interestingly, these shotgun data also reveal that the bacterial microbiome of bulk soil samples has a weaker but significant enrichment in several of these same categories (Fig. 2b), which was not observed previously in analysis of the metatranscriptomic data^6^. More generally, analysis of drought-induced shifts in functional capacity appears to show more similar patterns between rhizosphere and soil communities than was observed in prior analysis of metatranscriptomic activity, with six of ten significantly enriched categories in rhizospheres or soils appearing as enriched under both (Fig. 2a, b). This could be driven by the subtle but statistically significant drought enrichment in Actinobacteria reads in soil metagenomic datasets at TP8 (log_2_ fold change = 0.584, *p-*value = 7.65 × 10^−6^, Supplementary Fig. 4b, d), which was absent in previously published soil metatranscriptomics datasets^6^. Collectively, these data and prior results demonstrate that while differences exist between metagenomic and metatranscriptomic analyses in this system, several functional categories appear as signatures of both drought-enriched community functional capacity and drought-enriched transcriptional activity in the rhizosphere^6,9^.

### Assembly of 55 draft metagenome-assembled genomes (MAGs) from rhizosphere and soils

**Fig. 3:**
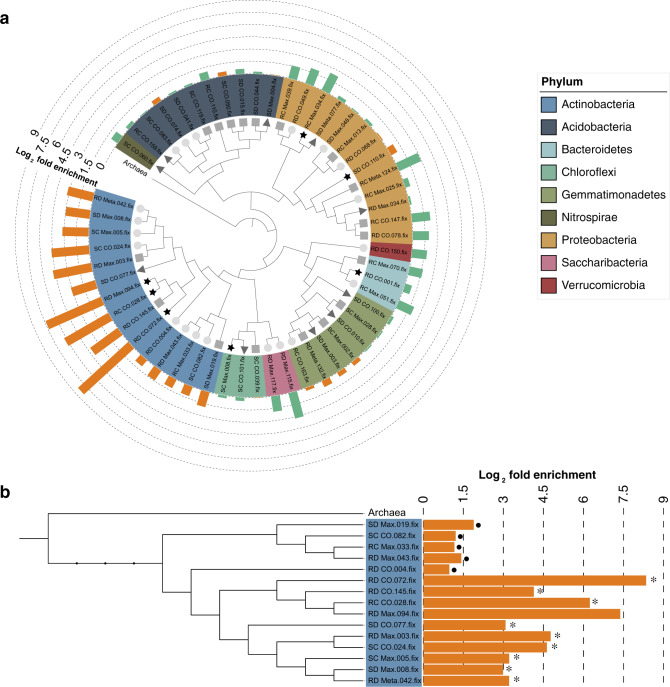
Phylogenetic tree of the 55 MAGs. **a** The phylogenetic tree at the center of the figure was constructed from all 55 MAGs. Symbols located at the end of tree branches (inner ring) represent the estimated genome completeness of each MAG. Stars (black) indicate completeness greater than 90%; triangles (dark gray) represent completeness between 80 and 89.9%; squares (medium gray) indicate completeness between 70 and 79.9%; circles (light gray) indicate completeness between 60 and 69.9%. The colored middle ring represents the phylum each MAG belongs to. The outer ring of colored bars represents the relative log_2_ fold enrichment (orange) or depletion (green) of each MAG within drought-treated rhizosphere compared with watered control rhizosphere. The tree was re-rooted using an Archaeal outgroup clade. **b** Phylogenetic tree of 15 Actinobacterial MAGs pruned from the tree of 55 MAGs in (**a**). The label on the left represents the name of each MAG in the dataset. The bar plot at the right indicates the log_2_ fold enrichment under drought in the rhizosphere community. Dots to the right of a bar indicate non-significantly enriched MAGs, *p*-value > 0.05; asterisks to the right of the bar indicate significantly enriched MAGs, *p*-value < 0.05. The log_2_ fold enrichment and *p* values were calculated using edgeR.

The analysis described above provides insight into community-wide functional capacity and activity within drought-stressed rhizospheres, but does not enable a direct comparison of function between specific drought-enriched and drought-depleted community members. To achieve this, we reconstructed genomes of rhizosphere and soil-associated bacteria by binning shotgun reads using cross-sample differential abundances to improve bin assignment^18^. In order to obtain longer contigs and lower species' heterogeneity, all samples were sorted prior to assembly into four groups based on each possible combination of sample type and treatment (control rhizospheres, drought-treated rhizospheres, control soils, and drought-treated soils), with each group containing samples from the same set of time points and identical numbers of replicates. After elimination of low-quality bins and potential duplicates across the four groups, 55 MAGs were obtained (Supplementary Fig. 3) with genome completeness estimates ranging from 60.0 to 99.1% (Fig. 3a) and estimated contamination levels ranging from 0 to 9.8%. Taxonomic assignment of these MAGs revealed bins belonging to the major root-associated phyla, including Actinobacteria, Proteobacteria, Acidobacteria, and Gemmatimonadetes, among others (Fig. 3a, Supplementary Fig. 7).

To determine how well these MAGs represent the drought-induced community compositional patterns within the broader experimental dataset, we mapped all shotgun read data from our study onto the 55 MAGs. Overall, 4.8% of reads could be mapped to the MAGs. This level is similar to what has been reported recently in genome-resolved metagenomic studies of other soil communities, 2.7−22.4% of the reads from each sample were recruited by the MAGs^27^. As was observed for the total co-assembled contigs derived from shotgun data (Fig. 1c, d), an analysis of Actinobacterial relative abundance in the MAG dataset across time and treatment revealed broad similarity to amplicon datasets, including enrichment of Actinobacterial abundance during drought treatment, enrichment during early sorghum development in control-treated rhizospheres, and a loss of enrichment in drought-treated samples following a rewatering period (Fig. 1e, f). Additional differences between the MAG datasets and the co-assembled contig analysis were also apparent, for instance, higher mapping to Chloroflexi, but are not unexpected given the incomplete collection of rhizosphere genomes for mapping within our assembled MAGs.

Further analysis of the relative abundance of Actinobacterial MAGs across treatments revealed differences in response to drought within the rhizosphere. While the relative abundance of all 15 Actinobacterial genomes increased under drought (Fig. 3b), only ten of the 15 Actinobacterial MAGs exhibited significant enrichment (average log_2_ fold change = 4.80, *p*-value < 0.05). The remaining five MAGs located in basal positions within the phylogenetic framework exhibited no significant enrichment (average log_2_ fold change = 1.33, *p*-value > 0.05, Fig. 3b). These data suggests that even within the phylum Actinobacteria distinct drought response phenotypes may exist. Based on these results, we hypothesized that a comparative genomics approach across these 15 Actinobacterial MAGs may help identify specific genetic differences that contribute to the strength of the enrichment phenotype.

### MAG-based comparative genomics identifies differentially abundant functions between enriched and nonenriched Actinobacteria

**Fig. 4:**
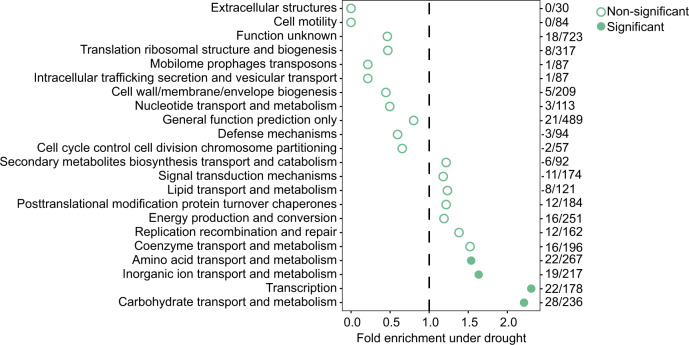
GO enrichment analysis of enriched Actinobacterial metagenome-assembled genomes (MAGs) at the peak of drought in the rhizosphere. GO enrichment analysis for all COGs showing enrichment within genomes of enriched Actinobacterial MAGs (*n* = 10) as compared with nonenriched Actinobacterial MAGs (*n* = 5) under drought for rhizosphere at time point 8 (TP8). The values on the *x*-axis indicate the fold enrichment ratio of the relative percentages of COGs upregulated under drought in each category relative to the total relative percentage of COGs in the corresponding category within the entire dataset. Solid (filled) circles indicate categories for which the enrichment had a *p*-value < 0.05 in a one-sided hypergeometric test and empty circles indicate categories with *p* values > 0.05.

To test this hypothesis, we performed gene predictions and annotations for all 55 MAGs using the Integrated Microbial Genomes database (IMG)^28^. A comparison of functional assignments between enriched (*n* = 10) and nonenriched (*n* = 5) Actinobacterial MAGs was then performed with the IMG-ER statistical analysis function using individual COG features. This analysis revealed 215 COGs that showed significant relative increase within the enriched Actinobacterial group (Supplementary Data 1), and 194 COGs that showed significant enrichment within the nonenriched Actinobacterial group (Supplementary Data 2). Consistent with the results from the previous functional analysis of co-assembled contigs, we observed within the enriched Actinobacteria group a significant relative increase of COG categories associated with carbohydrate transport and metabolism (Fig. 4, total COG count: 28). Additionally, we observed a significant relative increase in three other categories: amino acid transport and metabolism, transcription, and inorganic ion transport and metabolism (Fig. 4). Of those belonging to the carbohydrate and amino acid-related categories, many COGs were annotated with specific transport functions identified as drought enriched in our previous metatranscriptome study, including ABC transporters of glycerol-3-phosphate, proline/glycine betaine, and xylose^6^ (Supplementary Data 1). Notably, both of the other identified categories, transcription and inorganic ion transport and metabolism, were also identified as enriched under drought in the community-wide analysis of functional capacity (Fig. 2a). To explore the extent to which these enrichment patterns hold true outside the phylum Actinobacteria, a similar comparison of functional assignments between all enriched Actinobacterial MAGs (*n* = 10) and all other nonenriched MAGs (n = 45) across all phyla was performed as described above. This analysis revealed enrichment in many of the same categories, including all four described above, as well as several new ones (secondary metabolite transport and metabolism, lipid transport and metabolism, coenzyme transport and metabolism, and energy production and metabolism Supplementary Fig. 8). Collectively, this comparative analysis across small sets of MAGs with differential abundance across drought stress reveals both new and previously identified links between functional categories and drought enrichment status.

Interestingly, several recent studies have revealed connections between plant microbiome regulation and inorganic ion transport and metabolism, including those that have identified relationships between phosphate and iron homeostasis and microbiome regulation^29,30^. Additionally, other work has suggested that plant nutrient status may be directly perturbed by drought stress^31^. To explore the role that microbial functions related to inorganic ion transport and metabolism play in the observed drought-enrichment phenotypes, we further dissected COGs within this functional category. This analysis revealed that 15 of the 19 drought-stress-enriched COGs in inorganic ion transport and metabolism were specifically related to iron metabolism (Supplementary Data 1). These iron-related COGs include ABC-type cobalamin/Fe^3+^-siderophore transport systems (Mann−Whitney Test, *p-*value *=* 0.011), NADPH-dependent ferric siderophore reductases (Mann−Whitney Test, *p-*value *=* 0.005), Mn^2+^ and Fe^2+^ transporters of the NRAMP family (Mann−Whitney Test, *p-*value *=* 0.040), ferredoxins (Mann−Whitney Test, *p-*value = 0.002), predicted Fe^2+^/Mn^2+^ transporters (Mann−Whitney Test, *p-*value = 0.012), and YaaA cytoplasmic iron level regulating proteins (*p-*value = 0.011). Additionally, manual curation of the full set of 215 drought-enriched COGs yielded a significant number of additional COGs (*n* = 23) with functional annotations that did not cluster into the category inorganic ion transport and metabolism in our IMG-based analysis, but which are likely related to iron metabolism based on a survey of the scientific literature (Supplementary Data 1). Based on this result, derived entirely from a comparison within the phylum Actinobacteria, we hypothesize that organisms that encode a larger number of genes related to iron metabolism are more fit under drought conditions within the rhizosphere. Taken together, these results reiterate the potential importance of carbohydrate and amino acid transport, and implicate the new function of iron metabolism and transport, for microbial fitness in the drought-stressed rhizosphere.

### Host iron status

**Fig. 5:**
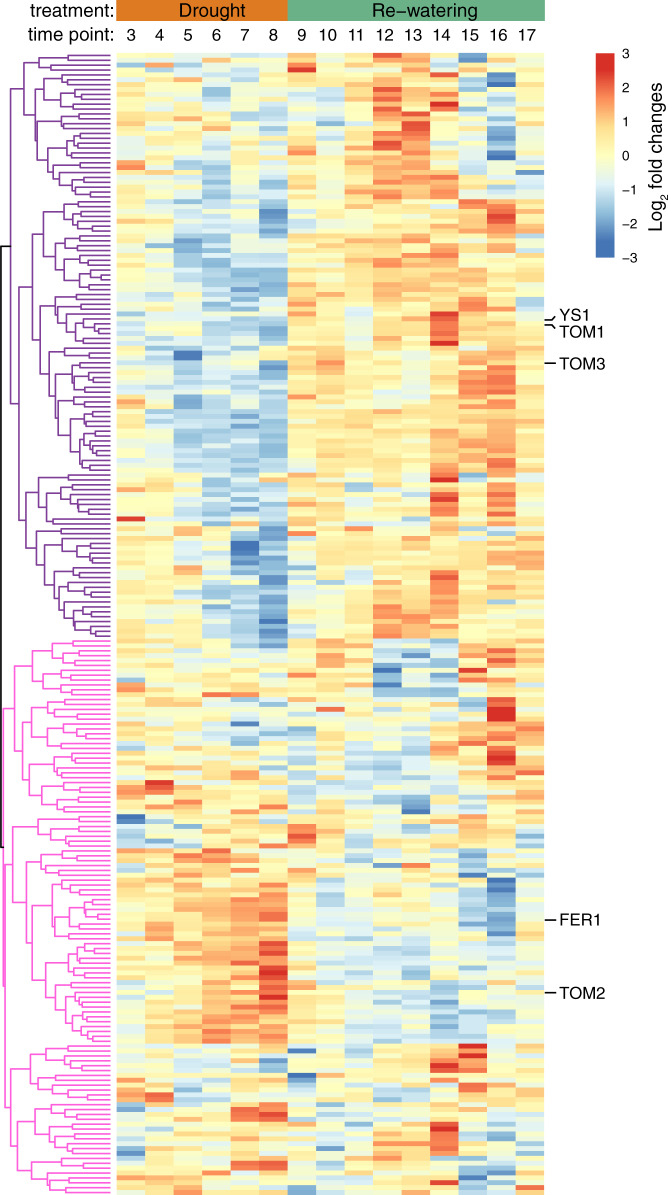
Iron metabolism gene expression in sorghum roots under drought stress. Log_2_ fold expression changes across time (*x*-axis) in drought-stressed root tissue for the approximately 234 expressed genes annotated as related to iron homeostasis (see Supplementary Data 3). Time points are indicated across the *x*-axis at the top, and drought (orange) and recovery (green) treatment stages are indicated above the time points. Hierarchical clustering analysis demonstrates two broad patterns of gene expression: a set of genes exhibiting strong downregulation under drought stress (indicated with purple tree branches), and a set of genes exhibiting strong upregulation under drought stress (indicated with pink tree branches). The five labeled genes on the right represent the genes investigated using qRT-PCR in the maize wild-type and *tom1* mutant in the next section.

Given the evidence that iron-related functionality is correlated with drought enrichment phenotypes in Actinobacteria, we hypothesized that iron homeostasis in the root system may be perturbed by drought. To explore this, we performed an analysis of host transcription using recently published RNA-Seq data^32^ that came from the same drought-stressed sorghum root samples used for the microbiome analysis described above. First, we identified within the Phytozome database^33^ a subset of 281 sorghum gene features expected to be involved in iron homeostasis based on annotation and homology to rice^34^, maize^35,36^, and Arabidopsis^37^ (Supplementary Data 3). Next, we extracted the log_2_ fold change in FPKM (fragments per kilobase of transcript per million mapped reads) values from root tissue samples between control and drought conditions across 15 time points (TP3−TP17), including the six time points (TP3−TP8) during which drought stress was imposed and nine time points (TP9−TP17) that fall within the subsequent rewatering treatment^32^. Expression profiles were available for 234 of the 281 putative iron-related genes in sorghum root, likely due to lack of expression of the remaining 47 genes in the tissue and environmental conditions surveyed in this study. In broad terms, hierarchical clustering of the resulting data across all 234 genes revealed two major clusters of gene expression (Fig. 5). In the first cluster (*n* = 120), which represented the majority of the genes, we observed a general trend of decreased expression levels under drought, with strong increases in expression levels upon re-watering. In the second cluster (*n* = 114), gene expression generally increased under drought stress and decreased upon re-watering (Fig. 5). Within the second cluster, a large number of genes are annotated as either iron−sulfur cluster binding domain-containing proteins or ferritins; ferritin is an iron-storage protein used in the storage sequestration of iron, while iron−sulfur cluster protein complexes are often involved in sensing and responding to oxidative stress^38,39^. By contrast, all of the iron transporter genes responsible for iron uptake from the surrounding soil belong to the first cluster and are strongly downregulated under drought. Collectively, these results suggest that iron homeostasis is disrupted by drought stress in sorghum roots, with a strong reduction in iron-uptake systems and an increase in iron-storage mechanisms.

### Loss of a host phytosiderophore transporter increases Actinobacterial abundance in the maize rhizosphere

Actinobacteria are known to make use of a variety of iron-uptake strategies, including some that are lineage-specific, and Gram-positives and Gram-negatives differ in their strategies for iron acquisition due to differences in the cell membrane and cell wall characteristics^40^. Given the observed decrease in iron-uptake strategies within the host during drought, and the enrichment of iron-metabolism-related functional capacity in drought-enriched Actinobacteria, we hypothesized that artificial reduction of plant iron uptake through use of plant genetic mutants may provoke rhizosphere microbiome shifts similar to those observed under drought, specifically an increase in root-associated Actinobacteria. To test this hypothesis, we first attempted to identify genes in plant iron-uptake pathways interfacing with the rhizosphere. Among the iron-uptake genes within sorghum roots that show strong downregulation at the peak of drought (TP8) is a homolog of a well-characterized iron phytosiderophore transporter, *TOM1* (Sobic.008G084800.v3.1, average log_2_FC = −5.7). TOM1 represents a class of exporters of the phytosiderophore mugenic acid (MA), which is used by the plant to chelate iron (Fe^+3^) in the surrounding soil environment for uptake back into the root system^41^. If reduced iron uptake under drought contributes to Actinobacterial enrichment, we hypothesized that under control condition mutants deficient in TOM1 functionality would exhibit increased Actinobacterial abundance. We further hypothesized that under drought conditions, little compositional shift would be observed in a *tom1* mutant, as *TOM1* is typically downregulated under these conditions.

As sorghum lacks well-characterized mutant libraries, we instead used loss-of-function mutants of *tom1* (previously identified as *ys3*^42^, GRMZM2G063306) in maize (MaizeGDB, https://www.maizegdb.org/gene_center/gene/GRMZM2G063306). Maize is a close relative of sorghum that has been shown to exhibit a similar drought-induced shift in the microbiome, including enrichment of Actinobacteria^11^. To validate that this previously characterized *tom1* line exhibits reduced root iron levels and altered expression of other iron-uptake genes in our experimental system, maize *tom1* mutants were grown in the growth chamber under both drought and control conditions in field soil collected from the site of our previous experiments. *qRT-PCR* with primers specific to *TOM1* revealed a loss of *TOM1* expression in the mutant background under control conditions as previously published (Supplementary Fig. 9). Several other genes involved in iron uptake (*TOM3*) and iron storage (*FER1*) also showed significant differences in the *tom1* mutant compared to WT under control conditions. Additionally, these data demonstrate that the pattern of response following drought treatment for all five genes tested in a WT maize background is consistent with trends observed in the sorghum transcriptomics data (Supplementary Data 3). Finally, ICP-MS analysis of root tissue in the WT and *tom1* mutant lines demonstrates that while WT maize exhibits a decrease in iron levels under drought stress, this difference is not observed in the *tom1* mutant background (Supplementary Fig. 10). These data indicate that TOM1 is required for the differential root iron levels observed across drought and control conditions.

**Fig. 6:**
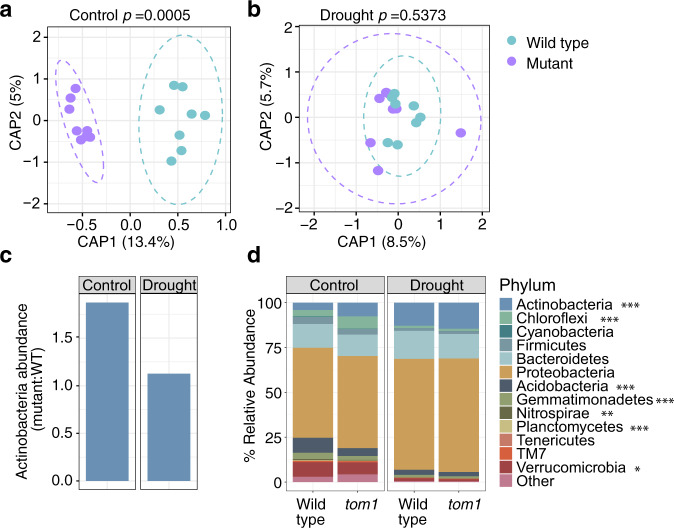
TOM1 deficiency in plants shapes the composition of the rhizosphere microbial community. Constrained ordination of rhizosphere microbiome composition showing the effect of plant genotype (wild-type or *tom1* mutant) under control (**a**) and drought (**b**) conditions. Ellipses show the parametric smallest area around the mean that contains 95% of the probability mass for each genotype. The *p*-value at the top of each plot represents the significance of the difference between WT and mutant, as evaluated by one-sided PERMANOVA. **c** The ratio of the relative abundance for Actinobacteria in *tom1* vs. wild-type maize under control (left bar) and drought (right bar) conditions. **d** Relative abundance for the 13 most abundant bacterial phyla in the rhizosphere in both wild-type and tom1 mutant plants grown in the growth chamber for either 2.5 weeks of control (left facet) or drought (right facet) conditions. Significant differences in abundance between WT and the tom1 mutant under control conditions were determined by paired two-sided T-test, and are indicated with asterisks to the right of the phylum name: *, *p* < 0.05; **, *p* < 0.01; ***, *p* < 0.001.

To test our hypothesis that loss of TOM1 would lead to altered microbiome composition similar to that observed under drought stress, bacterial rhizosphere microbiomes were profiled in the same experimental setup used for measuring *TOM1* expression and iron levels described above^6^. First, we validated that wild-type sorghum and maize rhizospheres share similar compositional responses to drought stress (Supplementary Fig. 11), including the expected increase in Actinobacteria. Second, we compared rhizosphere composition in the *tom1* mutant maize to WT maize under drought and control conditions. Consistent with our hypotheses, these data demonstrate that the rhizosphere microbiome of the *tom1* mutant is significantly different from wild type under control conditions (PERMANOVA, F-statistic = 4.9101, *R*^2^ = 0.2597, *p-*value = 0.0005, Fig. 6a), and that little change is observed between *tom1* and wild type under drought conditions (PERMANOVA, F-statistic = 0.9656, *R*^2^ = 0.0645, *p*-value *=* 0.5373, Fig. 6b). CAP analysis for all samples indicates that genotype explains 4.6% of the total variance, while under treatment, explains 53.4% (Supplementary Fig. 12). Next, we found that the relative abundance of Actinobacteria is significantly different between the *tom1* mutant and wild type in the rhizosphere under control conditions (fold change = 1.8, *p*-value = 0.00001, Fig. 6c, d), but this difference is diminished under drought (fold change = 1.1, *p*-value = 0.42798, Fig. 6c, d). Other lineages also exhibited changes in the mutant, including increases in Chloroflexi, and decreases in Acidobacteria, which are only partially consistent with changes observed under drought as compared to control in WT maize. Collectively, these data demonstrate that perturbation of iron metabolism-related genetic pathways in the root can impact rhizosphere composition, and that the observed shifts include changes in Actinobacterial abundance similar to those observed under drought.

### Exogenous iron disrupts the plant growth promotion capacity of *Streptomyces* under drought

**Fig. 7:**
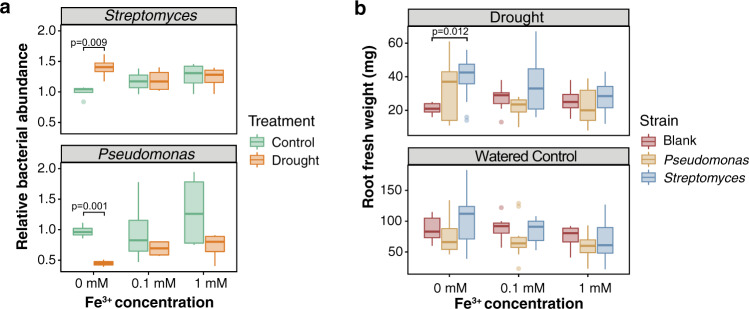
Exogenous iron disrupts Streptomyces enrichment and reverses plant growth promotion capacity. **a** Boxplot of the relative bacterial amount as measured using qPCR with lineage-specific primers across control (green) and drought-treated (orange) root samples in both Streptomyces ceolicolor Sc1 (upper panel) and Pseudomonas syringae (bottom panel) strains across two levels of iron application and a mock control (0 mM Fe3+). Values are means ± SD (*n* = 4) from four independent biological replicates. **b** Measurement of fresh root weight phenotypes upon application of two levels of Fe^3+^ and a mock control (0 mM Fe^3+^) causes a loss of root growth promoting phenotypes conferred by the Actinobacteria Streptomyces under drought (upper panel). No significant differences were observed under control conditions (lower panel). One-sided Tukey’s multiple-comparison tests were used for calculating the *p* values between different inoculations. Values are means ± SD (*n* = 7) from seven independent biological replicates. For both (**a**), (**b**), one-sided Tukey’s multiple-comparison tests were used for calculating the *p* values between different inoculations. Box bounds indicate one quartile above and below the mean, while whiskers indicate one standard deviation above the mean.

As TOM1 is a key player in iron import to the root, and as loss of TOM1 leads to an increase in Actinobacteria within the root, we hypothesized that reduced iron uptake within the root may promote Actinobacterial growth. We further hypothesized that the increased Actinobacterial abundance in roots that is typically observed under drought stress may be disrupted by exogenously applied iron. To test this, we performed an iron supplementation experiment in which drought, iron supplementation (mock, 0.1 mM and 1 mM Fe^3+^-EDTA), and microbial strains were applied to sorghum seedlings in a controlled, germ-free, growth chamber experiment. For microbial inoculum, individual sorghum root-derived strains of *Streptomyces coelicolor* Sc1 (a member of the phylum Actinobacteria) or *Pseudomonas syringae Ps1* (a member of the phylum Proteobacteria) that have been previously shown to be enriched and depleted under drought conditions, respectively, were applied independently^6^. To quantify bacterial abundance in the root, we performed *qPCR* using bacterial lineage-specific primers as previously described^6^. Consistent with previous findings, we observed that under mock conditions (0 mM iron supplementation), the absolute abundance of *Streptomyces Sc1* and *Pseudomonas Ps1* shows patterns of increased and decreased abundance, respectively, under drought as compared to control conditions (one-sided Tukey multiple comparisons of means, *Sc1*: *p*-value = 0.0093, diff = 0.4001, *Ps1*: *p*-value = 0.0006, diff = 0.5485, Fig. 7a). However, as hypothesized, the addition of exogenous iron disrupts these relationships, causing abundance levels during drought to remain similar to those observed under control treatment for both strains (one-sided Tukey multiple comparisons of means, all *p* values *>* 0.05, Fig. 7a). This result is consistent with a model in which iron limitation in the root promotes *Streptomyces* growth and hinders the growth of *Pseudomonas*. Interestingly, while we had previously observed that the addition of *Streptomyces* to drought-stressed, gnotobiotic grown sorghum led to increased plant root biomass^6^, here we observed that the addition of iron results in a loss of this stress-induced benefit (Fig. 7b). Additionally, we noted that plant shoot biomass did not show a significant increase under drought conditions with additional iron (Supplementary Fig. 13). Collectively, these results suggest that increased iron availability within the root environment influences plant−microbe interactions, reducing both the fitness advantage and plant growth promotion ability of *Streptomyces* under drought.

## Discussion

Whole-genome metagenomic sequencing provides tremendous value for microbiome studies, allowing for comparisons to the results obtained with more commonly used techniques and also analyses not possible with other data types^43^. In this study, metagenome data helped validate broad compositional shifts obtained through amplicon techniques in the same study^6^, corroborating previously described enrichment of Actinobacteria in drought-stressed roots observed in a variety of plant systems^6,9–11,14,15^. Additionally, these new analyses revealed data-type specific differences, for instance, overrepresentation of Proteobacteria and Actinobacteria, and the reduced representation of phylum Bacteroidetes, in metagenomic datasets. Similar discrepancies between amplicon and shotgun datasets from the same environmental samples have been noted in other recent studies^24–26,44^, and may be ascribed to biases found in amplicon data types, for example, reduced PCR and reference gene copy number-related issues^45,46^, or biases in shotgun datasets, such as those imposed by incomplete taxonomic representation in reference databases, taxonomic bias in gene annotation, and greater variability in analytical output from available analysis pipelines^43^. Analysis of microbiome composition through multiple methodologies can help identify these potential biases, and may help establish more accurate bounds on compositional shifts than possible through any single technique. Additionally, and more importantly, while amplicon data’s significantly reduced cost per sample typically allows analysis of a much larger number of samples, giving increased statistical power for measuring compositional shifts with amplicon datasets^43^ (Supplementary Data 4), metagenomic data allow for direct assessment of functional capacity that can only be imprecisely inferred from amplicon datasets. In this study, analysis of shotgun metagenomics data revealed that previously observed shifts in root microbiome activity for carbohydrate and secondary metabolite transport and metabolism during drought are driven at least partially by increased coding capacity for these pathways in drought-enriched lineages. It also revealed that drought-driven shifts in functional capacity differ less between soil and rhizosphere than do shifts in functional activity, suggesting that not all transcriptomic changes induced by drought in the rhizosphere are simply due to altered taxonomic composition of those communities.

In addition to providing insight into community-wide shifts in functional capacity, metagenomic sequencing also enables the reassembly of bacterial genomes from environmental samples for within-community comparative genomic analyses^47^. This culture-free approach, termed genome-resolved metagenomics^48^, has been successfully applied to probe microbiome function in multiple environments, including hospital rooms, the human gut, and oceans^17,49–51^, and has improved our understanding of the microbial world and its response to perturbation. Recent advances in binning of metagenomic datasets from complex environments now enable the reconstruction of uncultured bacterial genomes from soil microbiomes^18,52^, allowing for a dissection of soil-associated microbiome function with high taxonomic resolution. In this study, we applied this strategy to understand the underlying functional basis for drought-induced shifts in individual lineages within the rhizosphere microbiome. It represents one of the first studies performed in the plant rhizosphere to employ such a binning strategy^52,53^ and expands on prior successes by making use of paired host transcriptomics across a longitudinal transect of sorghum development and downstream reductionist experiments in controlled growth environments to help validate hypotheses generated through comparative analysis of MAG genetic capacity.

Our recovery of 55 bacterial genomes representing the most dominant phyla in the sorghum rhizosphere offers one clear advantage over traditional isolate sequencing, another standard method of functional assessment within a microbial community. This advantage is the ability to recover genomes from organisms that have been traditionally challenging to culture^48^. As part of this study, we recovered four Actinobacterial genomes classified within the class *Thermoleophilia*, a recently proposed lineage with very few species cultivated and characterized^54^; to date, there are only 18 reference genomes that belong to this class on the NCBI database (Taxonomy ID: 1497346), and none are closely related to the MAGs identified in this study. As all four of these MAGs were among our five nonenriched Actinobacterial genomes, a comparative analysis across enriched and unenriched lineages within this phylum would not have been possible using only currently available public reference data. It is worth noting that while only a fraction of the total shotgun dataset mapped to these 55 MAGs, broad trends in microbial composition and functional capacity observed in the complete co-assembled contig dataset were recapitulated in our analyses of the MAGs. We anticipate that future efforts with greater read depth and an increased number of MAGs will decrease discrepancies between these datasets, while enabling our ability to assign functional capacity to individual community members.

Unexpectedly, comparative genomics analysis across these Actinobacterial MAGs helped identify an intersection between drought stress enrichment phenotypes and iron metabolism. All living organisms on earth, with few exceptions, require iron to survive due to its inclusion as a co-factor in essential protein complexes^40^. Though iron is one of the most abundant elements in the crust of the earth, its availability to living organisms is limited by its oxidation state, solubility, and other environmental factors. To fulfill their needs for iron, all living organisms have highly specialized, and in some cases lineage-specific, systems to solubilize, transport, and store ferric (Fe^2+^) or ferrous (Fe^3+^) iron. Indeed, it has been shown that many Actinobacterial species secrete a variety of iron-chelating organic molecules that sequester and immobilize the solubilized form of iron (Fe^3+^) for uptake in the rhizosphere of plants growing in soil with low available iron^55^. A recent study of 277 isolated Actinobacteria strains belonging to 17 genera found that 39.4% display a capacity for reducing FeCl_3_ under oxic and initially neutral pH conditions, and living Actinobacterial cells and their metabolites play a key role in reducing Fe^3+^,^56^. More specifically, the genus *Streptomyces*, which is among the most highly enriched in the plant root community under drought stress^6,10^, is well-known to produce a variety of hydroxamate siderophores through divergent biosynthetic gene clusters^57^ that function to support growth in iron-limited environments. Interestingly, *Streptomyces* have recently been shown to gain a competitive growth advantage over other soil bacteria under low iron availability conditions^58^. A novel *Streptomyces* ‘exploration’ growth mode is triggered by low iron availability and is characterized by increased rates of colony expansion and greater production of iron-uptake strategies^58^.

We also demonstrate that plant roots undergo a significant change in iron homeostasis-related gene expression under drought stress. Indeed, root RNA-seq data demonstrate a downregulation of iron transporter genes and an upregulation of iron-storage functionality, which includes a set of ferritin genes. Data showing a perturbation of iron metabolism during the drought have been reported recently in Arabidopsis^59^, and Cantalapiedra et al. recently reported that genes related to the category ‘response to metal ion’ are enriched under drought in barley^60^. It is possible that downregulating iron uptake and upregulating iron storage may in fact be the plant’s strategy for dealing with drought-induced decreases in rates of photosynthesis^32^, as photosynthetic machinery represents one of the primary uses of iron in the plant. Alternatively, reduced iron uptake may represent the plant’s effort to protect cellular function in the root during heightened reactive oxygen species (ROS) activity^61^ that is known to accompany drought stress^62^. The combined presence of iron and ROS can trigger the Fenton reaction^63^, which produces superoxide radicals that are highly damaging to normal cellular function, modifying protein structures and functions via oxidative reactions involving protein thiol groups or iron-containing clusters^64^. Indeed, it is believed that these ROS species are, in fact, a likely cause of the primary pathologies observed in droughted plants^65^.

**Fig. 8:**
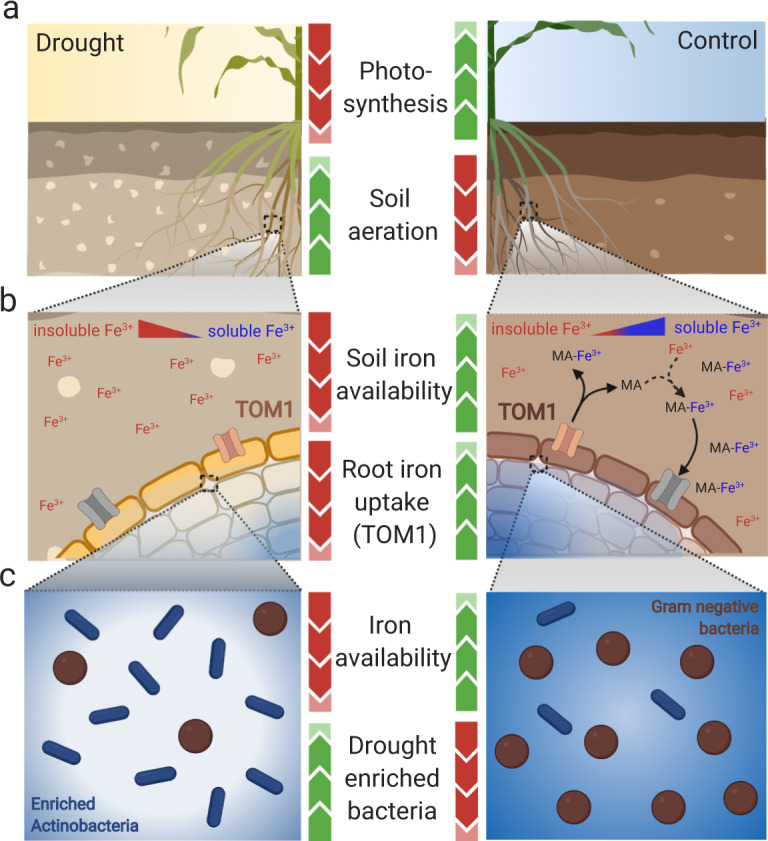
Model of interaction between iron, drought, and the root microbiome. **a** During drought stress, sorghum experiences a decrease in photosynthesis and consequent reduced need for iron uptake by the root. Simultaneously, the surrounding soil environment becomes increasingly aerobic as water is removed from soil pores. **b** Increased soil aeration leads to reduced iron availability for plants and microbes, as iron becomes increasingly stored as insoluble Fe^3+^ (shown in red text). Simultaneously, due to decreased need for iron and increased levels of ROS present within root tissues, sorghum roots decrease expression of iron-uptake machinery, including TOM1, which exports the phytosiderophore mugenic acid (MA) to the rhizosphere to solubilize Fe^3+^ (shown in blue text). Collectively, this leads to less-solubilized iron in the rhizosphere, and decreased available iron within the root compartment. **c** The resulting low iron availability in root and rhizosphere environments promotes the growth of drought-enriched bacterial taxa (shown in blue) with high copy number of iron transport and metabolism-related genes, which are able to better scavenge the limited iron than drought-depleted lineages (shown in brown). This figure was created using Biorender.com.

Prior work has suggested several potential mechanisms responsible for the observed drought enrichment of Actinobacteria within the root and rhizosphere, including both altered plant carbohydrate and amino acid metabolism and increased ROS production^6,16^. Here we propose that the enrichment within roots may also be partly explained by plant-driven decreases in iron availability within the root environment (Fig. 8). This is supported by evidence presented in this study, including: (1) reduced iron uptake by the sorghum root during drought, (2) the abundance of iron metabolism genes in enriched Actinobacteria, (3) induced Actinobacterial enrichment in a mutant maize line with reduced iron uptake, and (4) loss of Actinobacterial enrichment in drought-stressed roots upon application of iron. In this model, the root’s reduced iron uptake and increased iron storage leads to low available iron concentrations in the root as a whole. Bacteria that are able to more effectively scavenge the remaining iron thrive at the expense of those that cannot. The comparative genomics in our study suggests that this differential potential occurs not only between Actinobacteria and other phyla, but within the phylum Actinobacteria as well.

One remaining challenge is explaining Actinobacterial enrichment in the area outside the root. The data suggest that plant siderophore production and export to the rhizosphere decreases under drought stress, which should lead to greater iron availability there. One possibility is that the rhizosphere itself may experience a decrease in iron availability under drought due to other abiotic factors. Several studies have reported that drought does lead to decreases in available iron within soil environments^66–69^. This is consistent with what is known about iron availability and soil aeration; under highly aerobic conditions, which drought produces in soils (Fig. 8a), iron is oxidized to iron hydroxide (Fe(OH)_3_), which is poorly soluble in water, and not available for assimilation by both plants and microbes^70,71^. Alternatively, and perhaps more likely, it is known that bacteria living in the rhizosphere may partially rely on plant mechanisms of iron uptake for their own supply^72^. Indeed, there is evidence of intraspecies, interspecies, and even interkingdom, cheating strategies for iron uptake, in which some members of a community will import siderophores and reduce Fe^+3^ chelated by other organisms^73–77^. For instance, Pseudomonads have been shown to use a cheating strategy with the iron-scavenging molecule pyoverdine to acquire iron from hosts and other Pseudomonads^78^. Additionally, there are well-documented pathways by which *Pseudomonas*, *Bradyrhizobium*, *Bacillus*, and other soil bacteria are capable of taking up and utilizing fungal-produced ferrichrome siderophores such as ferricrocin^79,80^. Other studies have demonstrated that bacterial siderophores such as pyoverdine have a much higher affinity for iron than most phytosiderophores, including the TOM1-transported MA, allowing bacteria to remove iron first solubilized by and bound to phytosiderophores; this effectively makes phytosiderophores a good source of iron for bacteria^81,82^. Given the above findings, we propose that Actinobacteria enrichment under drought in the rhizosphere may be driven by a combination of reduced iron availability due to increased aerobic conditions in the soil, and reduced availability of plant phytosiderophores as a source of available iron for bacterial growth (Fig. 8b, c).

While these dynamics of iron competition and exchange have long been conjectured to be critical for shaping microbe−microbe and microbe−host dynamics^78^, it remains challenging to measure and assess them in the context of complex communities in and on host tissues, such as roots and rhizospheres. It is worth noting that other recent research has also identified a link between iron-related metabolism and the plant root microbiome in plants, primarily in eudicots. Voges et al. suggest that iron-mobilizing coumarins may influence the *A. thaliana* root bacterial community by means of inhibiting the proliferation of a relatively abundant *Pseudomonas* species, via a redox-mediated mechanism^83^. In addition, the protein MYB72 has been shown to shape the *Arabidopsis* root-associated microbial community by regulating the excretion of the coumarin scopoletin, an iron-mobilizing phenolic compound with selective antimicrobial activity^84^. Gu et al. established a causal mechanistic link between microbiome-level competition for iron and plant protection in tomatoes, and opened promising avenues to use siderophore-mediated interactions as a tool for microbiome engineering and pathogen control in plants^85^. Interestingly, the strategy used for iron uptake in eudicot plant models, such as Arabidopsis and tomato, is different from that used in monocots such as sorghum and maize. Eudicots use a reduction strategy involving alterations in soil pH to solubilize Fe^3+^, reduction of the Fe^3+^ via a membrane-bound ferric chelate reductase with subsequent uptake by Fe^2+^ transporters. Monocots use a chelation strategy, secreting phytosiderophores into the soil to bind Fe^3+^ and then use Fe^3+^-phytosiderophore transporters for uptake^86^. We anticipate that these differences could have important implications for interactions with the root microbiome, both under drought stress and under other environmental conditions.

The results of our study highlight a connection between iron metabolism, drought, and the plant root microbiome, and demonstrate the utility of adopting metagenome-guided comparative genomics for the discovery of such relationships. On the basis of these findings, we propose a rethinking of current approaches used to explore drought stress and its interactions with the microbiome in agricultural systems. We suggest that future studies of drought stress tolerance should be based on a perspective that takes into account relationships between plants, microbes, and nutrient status of the abiotic environment, especially as they pertain to models of the ability of plant microbiome interactions to modulate plant−water relationships.

## Methods

### Experiment design and sampling time points

In brief, the field experiments were conducted at the Kearney Agricultural Research Center in Parlier, California. Two sorghum cultivars (RTx430 and BTx642) were planted with a randomized block design and two different types of irrigation (drought stress and watered controls) with three replicate blocks per treatment^6^. Plant emergence was recorded on June 1, 2016, which coincides with TP0. All additional sequential time points represent the number of weeks past since plant emergence (eg., TP3 is exactly 3 weeks after TP0). In this study, rhizosphere and bulk soil samples were collected for shotgun metagenomics samples at TP3, 4, 8, 9, 10, and 11 (Supplementary Fig. 1). Sorghum transcriptomics data used in the generation of Fig. 5 were previously published in Varoquaux et al 2019^32^, and were generated for all time points between TP2 and TP17. The soil and rhizosphere sample collection strategy used is summarized below.

#### Irrigation management

Drought treatment was imposed on one-half of the blocks and consisted of a complete lack of irrigation until rewetting at the ninth week (TP9) after planting (WAP). Control blocks, which account for the remaining half of the blocks, received irrigation for the entire growing season beginning in TP3. The amount of water applied to control plants (TP3−TP17) and drought blocks (TP9−TP17) was 80% of the calculated evapotranspiration each week. Daily potential evapotranspiration (ET_o_) was determined from an on-site weather station located approximately 1200 m from the field site. A locally derived crop coefficient was matched to the crop-growth stage and multiplied by the ET_o_ values to determine a calculated daily estimated crop evapotranspiration (ET_c_) for a nonstressed grain sorghum crop. ET_c_ values were totaled for each Friday through Thursday 7-day period during the growing season and used to determine target irrigation amounts that matched 100% of ET_c_ to apply during each 7-day period. A drip system was utilized to apply all irrigation water during the growing season, consisting of drip lines placed on the surface of each furrow (0.76-m row spacing), with 0.3-m emitter spacing and 2 L/h emitter output. The drip system was only operated for part of one day out of each 7-day period, with treatments being irrigated for that period receiving an amount equivalent to 100% of the estimated ET_c_ calculated for the prior 7-day period. Surface drip lines were used for irrigation to provide accurate water application amounts and a high level of water application uniformity. The irrigation treatmernt designed to be a non-water-stressed control was irrigated at 7-day intervals between the first within-season irrigation (June 21) and the final irrigation (September 23). The drought stress treatment was imposed by providing no irrigation during the period from seedling emergence (June 3) until the flowering-growth stage, with the first within-season irrigations started on July 29 for that treatment. Irrigations continue after that date at timings and amounts that matched the nonstressed control treatments. Values of ET_o_ and ET_c_ are typical of those for summer-planted sorghum in the semiarid, relatively hot Mediterranean-type climate of the San Joaquin Valley. Measured rainfall during the June 3−September 29 period was less than 1 mm. As control and drought treatments were identical from the time of planting (T_0_) to the third week after planting (TP2), to avoid redundancy in sampling, drought treatment samples were only collected from TP3 through TP17.

#### Drought-treatment measurements

Percent depletion of ASM values shown represents the proportion of potentially plant-available stored soil water in the 0−30-cm zones of the soil profile (the same zone in which the roots and associated microbiomes were sampled) estimated using soil matric potential sensors placed at 15-cm depth. Soil matric potential measurements were made using Watermark sensors (Irrometer Corp, Riverside, CA), with sensors installed centered within plant rows, and all sensor placements replicated in three of the field replicas of plantings of each irrigation treatment (*n* = 6 for each treatment group). The relationship determined between matric potential and gravimetric water content for the experiment-site soil was used in combination with bulk-density measurements to determine volumetric soil water percent. Percent depletion of plant-available soil water on any date was determined (3) as the volumetric water content divided by the difference in volumetric water content between the estimated field capacity (−0.02 MPa soil matric potential) and permanent wilting point (−1.5 MPa soil matric potential).

#### Crop-water stress measurements

As a relative indicator of the degree of plant water stress and reduced water use associated with these soil water depletion periods, CWSI values were determined on select days during different growth stages in all three irrigation treatments using the approach outlined previously (*n* = 15 for each treatment group)^6^. CWSI has been described as a relative indicator of the levels of crop water stress, with values closer to 0.0 indicating non-water-stressed plants that transpire at levels indicating nonlimiting soil water availability, and a value of 1.0 indicating maximum water stress, with essentially no transpiration. For CWSI determinations, a handheld infrared thermometer (Everest Interscience, Tucson, AZ) was used to evaluate canopy temperature in 15 locations per field replication of each irrigation treatment during the period of 1400−1530 h PST. Air temperature and relative humidity were determined at a height of 2 m to match canopy temperature measurements, recorded on a data logger using air temperature and humidity sensors (Spectrum Technol., Aurora, IL) to allow determination of vapor-pressure deficit. Fully stressed and nonstressed baselines for the CWSI calculations were determined using in-field measurements collected during mid-afternoon periods representing vapor-pressure deficits ranging from 2.8 to 5.1 kPa.

#### Sample collection and processing

Plant samples were collected by manually extracting whole plants with root systems using a shovel to a depth of approximately 30 cm. We collected rhizosphere samples (soil tightly adhering to the sorghum root surface) by collecting and pooling roots severed with sterile blades from 10 plants per genotype and treatment type at each time point. Roots were vortexed in epiphyte-removal buffer (0.75% KH_2_PO_4_, 0.95% K_2_HPO_4_, and 1% Triton X-100 in ddH_2_O, filter-sterilized at 0.2 μM) for 5 min and centrifuged to pellet the resulting rhizosphere soil at 2000 x *g* for 5 min after removal of the root tissue. Root endosphere samples were obtained by washing the vortexed roots twice in a fresh sterile buffer and quick-frozen in liquid nitrogen. Bulk soil samples were collected approximately 12 inches away from the sample plants we collected using a 6-soil corer. All samples were collected weekly at the same time of day (between 10 am and 1 pm) and the same day of the week for 17 weeks following seedling emergence (TP1−TP17, Supplementary Fig. 1). To aid in the DNA extraction process, roots samples were first ground in a cryogenic Freezer Mill (SPEX SamplePrep 6875D, Metuchen NJ USA). For younger roots, the settings were 10 cps (cycles per second), 5 min of pre-cool, 2 cycles of 2 min of grinding, and 1 min of cooling in-between. For weeks 6−10 old roots, the settings were 10 cps (cycles per second), 5 min pre-cool, 3 cycles of 2.5 min grinding, and 1 min cooling in between. For 11 weeks or older, the settings were 10 cps (cycles per second), 5 min of pre-cool, three cycles of 3 min of grinding, and 1 min of cooling in-between.

#### DNA and RNA extraction

For the soil RNA extractions, as the MoBio PowerMax (Catalog No. 12988-10) and PowerSoil kit use the same silica membrane, we used the PowerMax soil DNA isolation kit for RNA extraction with a modified protocol provided by MoBio. Briefly, 3 g of soil and 5 ml of phenol:chloroform:isoamyl alcohol (25:24:1, pH 7−8) were added to the bead tubes with bead solution. To this, 10 ml of bead solution, 1 ml of solution C1, and 0.5 ml of solution C2 were added, and the tubes subsequently homogenized using a vortexer for 15 min. After centrifugation at 8000 g for 15 min, 3 ml of solution C3 was added to the supernatant and the tubes incubated at 4 °C for 5 min. The tubes were then centrifuged at 8000 g for 15 min, and the supernatant was transferred onto the Max column and the flow-through was discarded. The columns were washed three times with a wash buffer and then allowed to air-dry for 10 min inside a fresh tube. The RNA was eluted in 6 mL of solution C6 and centrifuged at 3000 g for 5 min. In all, 262 μl of 5 M NaCl was added to achieve a final concentration of 0.2 M. In all, 2.5 volumes of 100% ethanol were added, and the tubes were incubated at −20 °C overnight. Subsequently, the tubes were centrifuged for 20 min at 8000 g. The subsequent RNA pellet was washed with 70% ethanol and resuspended in 100 μl RNase-free H_2_O. The remaining DNA was digested using a DNase Max Kit (MoBio, Carlsbad, CA, USA) according to the manufacturer’s protocol and the RNA was purified using a RNeasy PowerClean Pro Cleanup Kit (MoBio, Carlsbad, CA, USA). The concentration was assessed using a Qubit 3 Fluorometer (Invitrogen, Carlsbad, CA, USA) and quality was assessed using an Agilent Bioanalyzer 2100 (Agilent, Santa Clara, CA, USA). For the qPCR experiments, plant root total RNA was extracted using the RNeasy Mini Kit (Qiagen Catalog No. 74104). A sample of 2 μg of total RNA was treated with DNase I (Invitrogen catalog No. 18068015), and reverse transcription was conducted using Superscript III First-Strand Synthesis System (Invitrogen Catalog No. 18080051) with oligo (dT) 18 primers. The quality of the cDNA was tested by qPCR with an internal control gene GAPDH (8). By the Ct (cycle threshold) value of the test run, we were able to normalize the amount of cDNA between samples.

#### Library preparation

For 16S library preparation presented here and published in ref. ^6^, DNA concentrations were measured with a Qubit 3 Fluorometer, and samples were diluted to 5 ng/μl to help ensure approximately equal template amounts, which we have found improves PCR yield. Samples were amplified using a dual-indexed 16s rRNA Illumina iTags primer set specific to the V3−V4 region (341F (5′-CCTACGGGNBGCASCAG-3′) and 785R (5′-GACTACNVGGGTATCTAATCC-3′) as described in (9) using 5-Prime Hot Master Mix (catalog No. 2200410). The reactions included 11.12 μl of DNase-free sterile H_2_O, 0.4 μg of BSA, 10.0 μl of 5-Prime Hot Master Mix, 2 μl of template, and 0.75 μM of each of two PNAs (4) designed to target host-derived amplicons from chloroplast and mitochondria 16S rRNA sequences. PCR reactions were performed in triplicate in three individual thermocyclers (to account for possible thermocycler bias) with the following conditions: initial 3-min cycle at 94 °C, then 30 cycles of 45 s at 94 °C, 10 s at 78 °C, 1 min at 50 °C, and 1.5 min at 72 °C, followed by a final cycle of 10 min at 72 °C. Triplicates were then pooled (192 samples per library) and DNA concentration for each sample was quantified using Qubit 3 Fluorometer. Pools of amplicons were constructed using 100 ng for each PCR product. Before submitting for sequencing, pooled samples were cleaned up with 1.0 × volume Agencourt AMPureXP (Beckman-Coulter, West Sacramento, CA) beads according to the manufacturer’s directions, except for the modifications of using 1.0 × rather than 1.6 × volume beads per sample, dispensing 1500 μl of 70% EtOH to each well rather than 200 μl, and eluting in 100 μl of DNase-free H2O rather than 40 μl. An aliquot of the pooled amplicons was diluted to 10 nM in 30 μl of the total volume before submitting to the QB3 facility at UC Berkeley for sequencing using Miseq 300-bp pair-end with v3 chemistry. The resulting amplicon libraries produced on average approximately 45259, 41582, and 34649 reads per sample for soils, rhizospheres, and roots, respectively. The use of PNAs reduced non-bacterial read contaminants to <3% in all sample types. For preparation of the metatranscriptome libraries from soil, a DNase Max kit (Qiagen, Catalog No. 15200-50) was used to digest DNA from the total RNA. Next, we used the Ribo-Zero rRNA Removal Kit (Bacteria, Illumina, Catalog No. MRZB12424) to remove ribosomal RNA from bacteria by following the manufacturer’s instructions. Subsequently, the TruSeq Stranded Total RNA Library Prep plant Kit (Illumina, Catalog No. 20020610) was used according to the manufacturer’s instructions to make 300−500 bp fragment libraries for sequencing on a HiSeq 2500 platform 150-bp paired-end. All of our sequencing was performed by QB3- Berkeley Functional Genomics Laboratory (http://qb3.berkeley.edu/fgl/).

#### Metagenomics

In order to improve the read depth and contig quality for assembling, additional samples collected as part of a post-flowering drought treatment in the original experimental design^6^ but not used in this study were processed and included for the assembly and binning process. As prior research revealed that post-flowering drought stress has a minimal impact on microbiome development compared to drought-imposed preflowering, these samples were not included for any additional measurements of analyses in this study outside of the genome-resolved metagenomic benign process. In total, 26 soil and 26 rhizosphere samples were collected across all time points (TPs) and treatments. The samples were immediately flash-frozen in the field with liquid nitrogen and maintained at −80 °C until nucleic acid extraction.

### Metagenomic assembly, gene prediction, and function annotation

A total of 680,999,720 read pairs of raw metagenomic reads were obtained. FastQC was used to assess the read quality^87^. Raw sequence reads were stripped of adaptor and low-quality base pairs and reads were trimmed/removed using cutadapt 1.6^88^. In total, 679,065,496 retained paired reads were assembled with megahit v1.1.2^89^ on a 120-core intel cluster node with 1.5T of RAM with k-mer 31−221. We performed two types of assembly. The first strategy is a co-assembly with a pool of reads from all samples (the first strategy of assembling). The de novo assembly was performed with megahit v1.1.2^89^. In total, we retrieved 49,816,847 contigs with 29,639,429,852 base pairs. An average GC content is 64.84%, N50 is 599, and L50 is 13,296,981. The contigs greater than 1,000 bp were used to perform the CDS prediction and function annotation with Prokka v1.13^90^. We predicted 9,484,562 protein-coding sequences (CDSs) from the contigs greater than 1,000 bp. All of the CDSs predicted from co-assembled contigs were assigned taxonomy with Kaiju v1.6.3^91^, resulting in 1,391,563 CDSs that could be identified as belonging to bacteria; these were extracted and used to estimate per-sample bacterial community composition. The read coverage for each gene at TP8 (the peak of drought) was estimated by kallisto^92^, and the differential coverage across treatments for each individual gene and COG category was calculated using edgeR v3.24.3^93^. Following this, a hypergeometric test was performed, as previously described^6^ to identify broad functional categories that have a significant overrepresentation of drought-enriched COGs. The second assemble strategy is presented in the genome-binning section.

### Community composition and function analysis

The 16S rRNA gene bacterial community profile was generated from the 16S rRNA gene amplicon sequencing reads^6^ and was processed using the iTagger v2.0.0 pipeline developed at the Joint Genome Institute^94^. The read counts were clustered into operational taxonomic units (OTUs) at 97% identity. Taxonomies were assigned to each OTU using the RDP Naïve Bayesian Classifier v2.9.0^95^. The CDSs from co-assembled contigs (first strategy of assembling) were assigned to taxonomy by running kaiju v1.6.3. The relative abundance of CDSs was estimated by Kallisto v0.42.4^92^ by mapping raw reads from each sample to CDS sequences. Reads belonging to each phylum within a sample were summed and calculated the relative abundance. The composition profile of shotgun metagenomic data for each sample was plotted in R. The differential expression level between different treatments for CDSs was calculated by edgeR v3.24.3. The CDSs were clustered to different pathways based on the COG database. A hypergeometric test was used to check the enrichment status of pathways and COG categories.

### Genome binning, dereplication, and taxonomy assignment

In order to reduce the contig heterogeneity for the binning process and while maintaining enough reads to retrieve long contigs, we grouped and performed the second type of co-assembly with megahit v1.1.2 for four groups of samples: bulk soil under drought, bulk soil under control, rhizosphere under drought, and rhizosphere under control samples, respectively. We filtered and kept the contigs ≥ 1 kb for binning with softwares CONCOCT v1.1.0^96^, metaBAT v2.12.1^97^, and MaxBin 2.0^98^. The best bin across the output of the three binning software results for each sample was selected using DAS_Tool v1.1.1^99^. Further, dereplication across all bins was performed with dRep v2.0.5 nucleotide^100^ according to average nucleotide identity (AN ≥ 99%). The resulting MAGs were assessed for completeness and contamination using CheckM v1.0.11^101^. Sixty-four MAGs satisfied the conditions of medium-quality genome (completeness > 50%, contamination < 10%) and seven MAGs showed high quality (completeness > 90% and contamination < 10%). We used the fifty-five genomes with the quality completeness > 60% and contamination < 10% for further analysis. The taxonomy assignment was predicted with CheckM v1.0.11^101^ and GTDB-Tk v1.3.0^102^. A manual curation was performed to combine the phylogenetic results from these two software packages. We only kept the taxonomy level that shows consistency between the two software packages. The multiple-sequence alignment file, which was generated by GTDB-Tk v1.3.0, including 55 MAGs and over 20,000 guide genomes, was used for building a phylogenetic tree. An archaea genome was added for tree re-rooting.

### Differential abundance analysis between the treatment for MAGs

Differential abundance of MAGs between control and preflowering drought treatment was determined using raw-read count data as the input. We used MetaBAT v2.12.1 with default parameters to map the clean reads from each sample back to MAGs. Based on the abundance profiles, the MAGs with significant differential abundance between treatments at the peak of drought (TP8) were determined using edgeR^93^. As the host genotype did not shape the bacterial composition in a significant way, we combined samples from different genotypes at the same time point and treatment as replicates^6^. Differential abundance of MAGs was tested by quasi-likelihood F-tests in edgeR v3.24.3. The resulting *p-*values <0.05 were considered as significant differences between treatments.

### Functional annotation and genome comparison

The CDS prediction and function annotation for co-assembled contigs of all samples was performed by Prokka v1.13^90^. All of the CDSs, which belong to bacteria and have a COG assignment, are filtered for further analysis. The expression level for each CDS was estimated by kallisto v0.42.4^92^ by mapping raw reads from 52 samples to each CDS. Differential expression patterns for CDSs were calculated by R package edgeR v3.24.3^93^. Hypergeometric tests were used to test each COG category that includes a group of significant differential expressed CDSs^6^. The gene prediction and annotation of MAGs were performed with IMG/ER^28^. The function comparison of two groups of genomes was performed with IMG by the ‘Statistic Analysis’ function. The parameters we used here are as follows: feature type: COG, measurements: gene count, statistical method: default and relative.

### Exogenous iron-solution treatment

The bacterial inoculation solution and the iron solution were mixed with soil in a laminar flow hood right before planting^6^. The two isolates we used were *Streptomyces ceolicolor* (*Sc1*) and *Pseudomonas syringae* (*Proteo*), which were isolated from the roots of drought-treated Sorghum bicolor RTx430 at 8 weeks old from sorghum present in our field at the Kearney Research and Extension Center, Parlier, CA.^6^. The Fe^3+^-EDTA solution was applied at two concentrations (0.1 mM and 1 mM). In all, 1 mM EDTA solution was used as the mock control. Sorghum cultivar RT430 was planted in our sterilized microbox system^6^. In brief, sorghum seeds were surface-sterilized and grown in our microboxes for four weeks until plants were harvested for quantifying bacteria amount and measuring plant phenotypes. The drought treatment (no water applied) was started when sorghum was 1.5 weeks old, and lasted for 2.5 weeks. The control plants were watered every five days with 350 ml of water/pot. The plants grew in a walk-in growth chamber under 16 h of light at 33 °C / 8 h of dark at 28 °C, with 60% humidity for four weeks before harvesting. The sorghum fresh root and shoot weight comparison between different inoculations and iron concentrations was evaluated by a post hoc test with TukeyHSD function in R.

### Bacterial quantification from microboxes

Quantitative reverse transcription−PCR was performed using QX200 EvaGreen Supermix (Bio Rad, Catalog No. 1864034). Three biological replicates were performed for each reverse transcription-PCR experiment. The Sorghum GAPDH gene is used as the internal reference to normalize the expression data (GAPDH-f: 5′-AAGGCCGGCATTGCTTTGAAT-3′, GAPDH-r: 5′-ACATGTGGCAGATCAGGTCGA-3′). The primers used for *Streptomyces* quantitation are Actino235 (5′-CGCGGCCTATCAGCTTGTTG-3’) and Eub518 (5’-ATTACCGCGGCTGCTGG-3’), and the primers for *Pseudomonas* quantitation are Eub338 (5′-ACTCCTACGGGAGGCAGCAG-3′) and Bet680 (5′-TCACTGCTACACGYG-3′). Relative expression levels were calculated according to the 2 − 2∆∆CT (cycle threshold) method^103^, and the standard deviation was calculated among the three biological replicates. The PCR conditions for the amplification were 95 °C for 2 min, and 50 two-step cycles (95 °C for 10 s, 60 °Cs for 25 s) followed by a plate read; a melt curve was generated by heating from 72 to 95 °C with 0.2 °C increments. The starting DNA template amount for taxa-specific *qPCR* is 10 ng per sample. The *p*-value of the relative bacterial amount between treatments under each condition was calculated by function t.test in R.

### *tom1/ys3* maize mutants

Maize *ys3-ref* allele previously identified as a loss-of-function mutant of maize *tom1* ortholog^104^ was acquired from Maize Genetics COOP stock center (311F)^104^. Segregating individual plants from a test-cross family were genotyped, and homozygous wild-type and mutant siblings were isolated for the rhizosphere sampling. We collected soil from the field plot where we performed our main experiment^6^. The soil was prepared with the following recipe per pot: 200 ml of tap water, 500 g of field soil, 400 g of calcined clay, and 100 g of vermiculite. The plants grew in a walk-in growth chamber under 16 h of light at 33 °C / 8 h of dark at 28 °C, with 60% humidity for four weeks before harvesting. The control plants were watered every five days with 350 ml of water/pot. The drought plants were watered every ten days with 200 ml of water/pot. Seven replicates per genotype and treatment were performed. Six unplanted pots filled with soil were used as a blank control, which was also equally separated into two groups of well-watered control and drought treatment. Rhizosphere was collected from each plant as described above. All of the samples were used for 16S sequencing as described above. Root total RNA was isolated using Trizol. For qRT-PCR analysis, cDNA was synthesized from DNase I-treated total RNA by SuperScript^®^ III First-Strand Synthesis System. Standard deviation was calculated among at least three biological replicates for each sample. The maize *GAPDH* gene was used as the internal reference to normalize the expression data^105^. All of the primers used for qPCR have been listed in Supplementary Table 1.

#### Root tissue collection

Root tissue was collected as previously described above. After isolation, roots were vortexed to remove rhizosphere soil, then rinsed twice in the root washing buffer, the same as the epiphyte removal buffer. The washed roots were then gently dried, placed in aluminum foil bags, and quickly frozen in liquid nitrogen. Each week, all samples were collected at the same time of day (between 10 am and 1 pm) and the same day of the week for 16 weeks following seedling emergence (Week 2−Week 17).

#### RNA extraction and sequencing

Harvested and frozen root and leaf tissue were ground in a cryogenic Freezer Mill (SPEX SamplePrep 6875D, Metuchen NJ USA) for 2−3 cycles of 2−3 min, with 1 min of cooling in-between. Samples were then stored at −80 °C. RNA was extracted using the QIAGEN miRNeasy Mini Kit (Cat. #AM217004) with modifications: 1 mL of QIAzol (Cat #AM217004) and 100 µL of 10% Sarkosyl solution were added to 100 mg of frozen tissue and placed in a vortex shaker adapter for 5 min. To these, 200 µL of chloroform was added, and the samples were then vortexed and incubated for 3 min at room temperature before centrifugation at 12,000 × *g* for 15 min at 4 °C. The aqueous phase was then transferred to a fresh tube, and ethanol was added to a final concentration of 60%. The remaining procedure was performed according to the kit manufacturer’s recommendations. DNA contamination was removed from each sample using the TURBO DNA-free kit (Cat. #AM1907, Invitrogen) according to the manufacturer’s recommendations. Stranded cDNA libraries were generated using the Illumina Truseq Stranded RNA LT kit.˙mRNA was purified from 1 µg of total RNA using magnetic beads containing poly-T oligos ˙mRNA was fragmented and reversed-transcribed using random hexamers and SSII (Invitrogen) followed by second-strand synthesis. The fragmented cDNA was treated with end-pair, A-tailing, adapter ligation, and 8 cycles of PCR. qPCR was used to determine the concentration of the libraries. Libraries were sequenced on the Illumina HiSeq.

#### Transcriptomic sequence data analysis pipeline

Raw fastq file reads were filtered and trimmed using the JGI QC pipeline resulting in the filtered fastq file (*.filter-RNA.gz files). Using BBDuk v 38.88(bbd), raw reads were evaluated for artifact sequence by kmer matching (kmer = 25), allowing 1 mismatch, and the detected artifact was trimmed from the 3′ end of the reads. RNA spike-in reads, PhiX reads, and reads containing any Ns were removed. Quality trimming was performed using the phred trimming method set at Q6. Finally, following trimming, reads under the length threshold were removed (minimum length 25 bases or 1/3 of the original read length—whichever is longer). Filtered reads from each library were aligned to the reference genome (phytozome v3.1, supplemented with RTx430 and BTx642 SNP information) using HISAT version 2.1.0 (BAMs/directory). featureCounts v1.22.2^106^ was used to generate the raw gene counts (counts.txt) file using gff3 annotations. Only primary hits assigned to the reverse strand were included in the raw gene counts (-s 2 -p—primary options). The statistical analysis methods used to calculate log_2_ fold change and *p* values of gene expression between control and drought conditions are described in detail in our previous study^32^. The expression heatmap of the iron-related genes was built with the pheatmap package v1.12.0 in R^107^.

### Statistics and reproducibility

All experiments were performed a single time, unless otherwise noted in the text.

### Reporting summary

Further information on research design is available in the Nature Research Reporting Summary linked to this article.

## Supplementary information

Supplementary Information

Supplementary Data 1

Supplementary Data 2

Supplementary Data 3

Supplementary Data 4

Reporting Summary

## Data Availability

The sequences reported in this paper have been deposited in the National Center for Biotechnology Information Sequence Read Archive, www.ncbi.nlm.nih.gov/ (accession nos. PRJNA435634).

## Code availability

The analysis code that supports the findings of this study is available at GitHub https://github.com/LingXu2018/EPICON_IRON.
