# Supplementary material for: Genome-resolved metagenomics reveals role of iron metabolism in drought-induced rhizosphere microbiome dynamics
Source: Nat Commun. 2021 May 28;12:3209. doi: 10.1038/s41467-021-23553-7 (PMC8163885; doi:10.1038/s41467-021-23553-7)
Supplement: Supplementary file 1 — Supplementary Information [file 41467_2021_23553_MOESM1_ESM.pdf]

Supplementary Figures

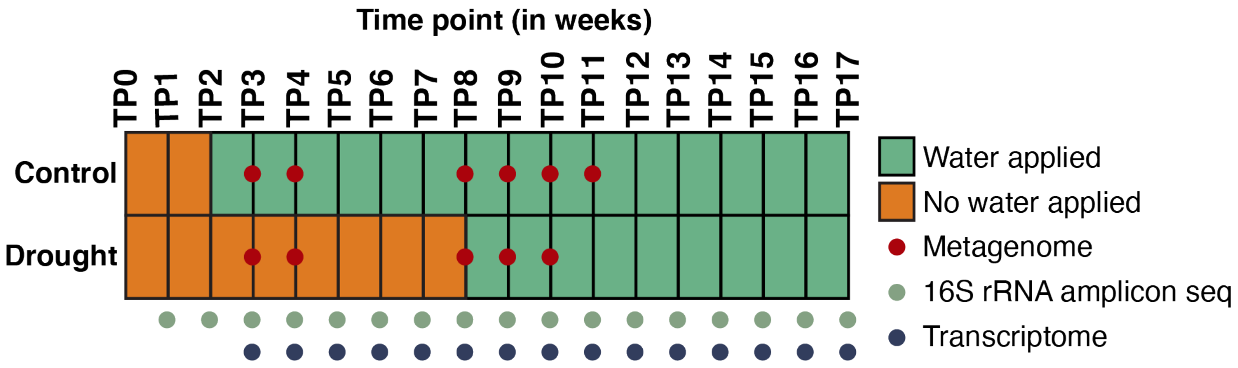

Supplementary Figure 1. Sampling design and timeline. Schematic diagram of the sampling design across time points, shown on the (x-axis). Sampling and watering times across the two experimental treatments are indicated within the two rows, with watered control treatment on the top row, and drought-treated treatment on the bottom row. Each box represents one week; green color within a block indicates a week in which plants were watered for a given treatment, whereas orange color within a box indicates weeks in which no water was administered. The amount of applied water in the watered control treatment varied by time points and was determined by supplying 80% of daily potential evapotranspiration (ET_o) at each phase of the project (see Methods). The red, green, and blue circles (located within and below the plot) represent weeks in which the shotgun metagenomics, 16S rRNA amplicon sequencing, and transcriptomics were performed, respectively.

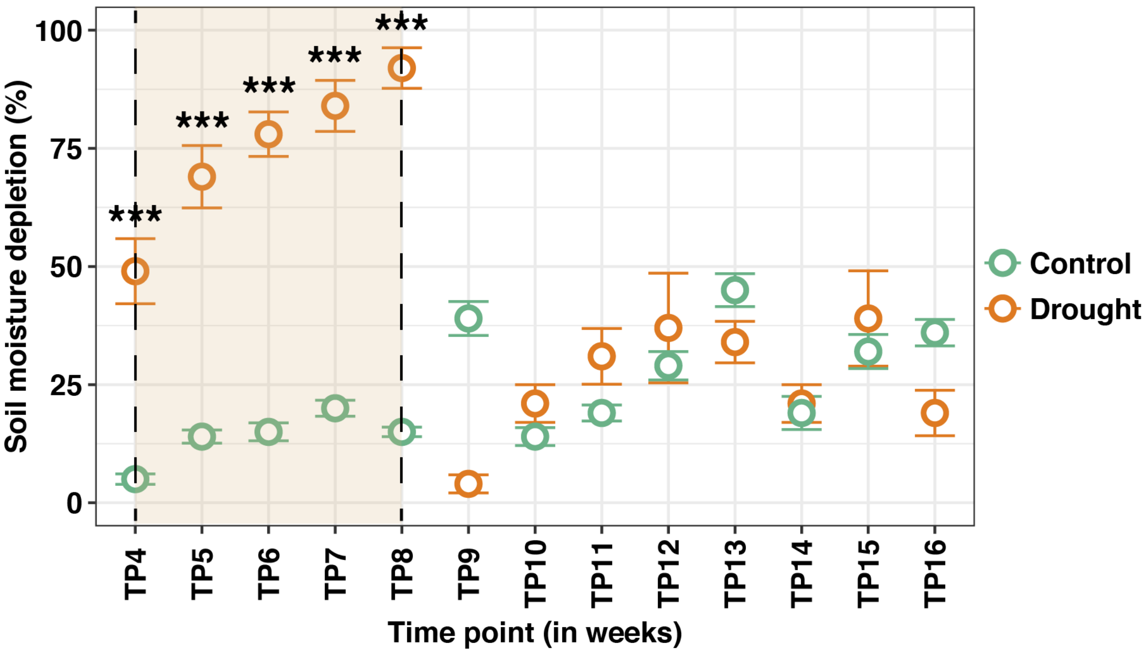

Supplementary Fig 2. Percent depletion of available soil moisture across the experimental timeline. Percent depletion of available soil moisture was estimated by collecting soil at 15 cm depth weekly from TP4 through TP16 throughout the experiment and calculating volumetric water content in each sample as described in the text (values are means ± SD (n = 6) from 6 independent biological replicates, see Methods). The orange shaded region demarcated by vertical dashed lines between TP4 and TP8 represents the period in this plot during which drought was applied (TP3 is not shown in this plot, but drought was also applied during this period). The green and orange circles within the plot represent mean values of percent depletion of available soil moisture across watered control and drought treatments, respectively. The whisker bars extending outside of each circle represent one standard error above and below the mean. To determine if percent depletion of available soil moisture differed between control and each drought treatment, a two-sided two-sample t-test was performed. Differences between drought and control during the drought period are all significant with a p value < 0.05, as indicated above each set of boxes within the orange shaded region (TP4-8, *** indicates p value = 0.0).

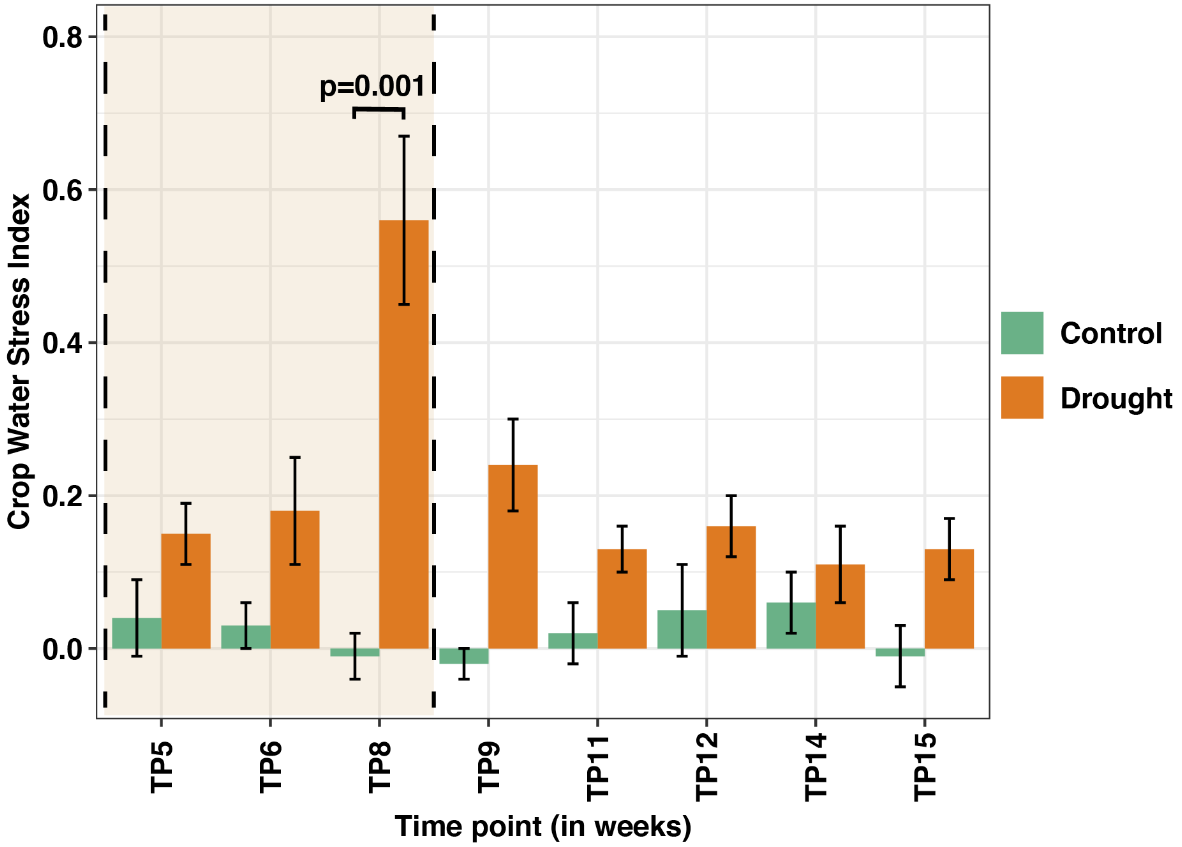

Supplementary Figure 3. Crop water stress index. Measurement of the Crop Water Stress Index (CWSI) were determined using a hand-held infrared thermometer (values are means ± SD (n = 15) from 15 independent biological replicates, see Methods) on select days during different growth stages in both irrigation treatments as a relative indicator of levels of crop water stress, with values closer to 0.0 indicating plants that transpire at normal levels and suggest non-limiting soil water availability, and a value of 1.0 indicating maximum water stress, with essentially no transpiration occurring. Green and orange bars indicate control and drought, respectively. The whisker bars at the top of each bar represents one standard error above and below the mean. The orange shaded region demarcated by vertical dashed lines indicates the period in this plot during which drought was applied (time point 3 (TP3) and TP4 are not shown in this plot, but drought was also applied during these TPs). To determine if CSWI differed between control and drought treatment, a two-sided T-test were performed. Significant differences between drought and control ($p<0.05$) are indicated with p values located above each set of boxes within the orange shaded region (TP5-8).

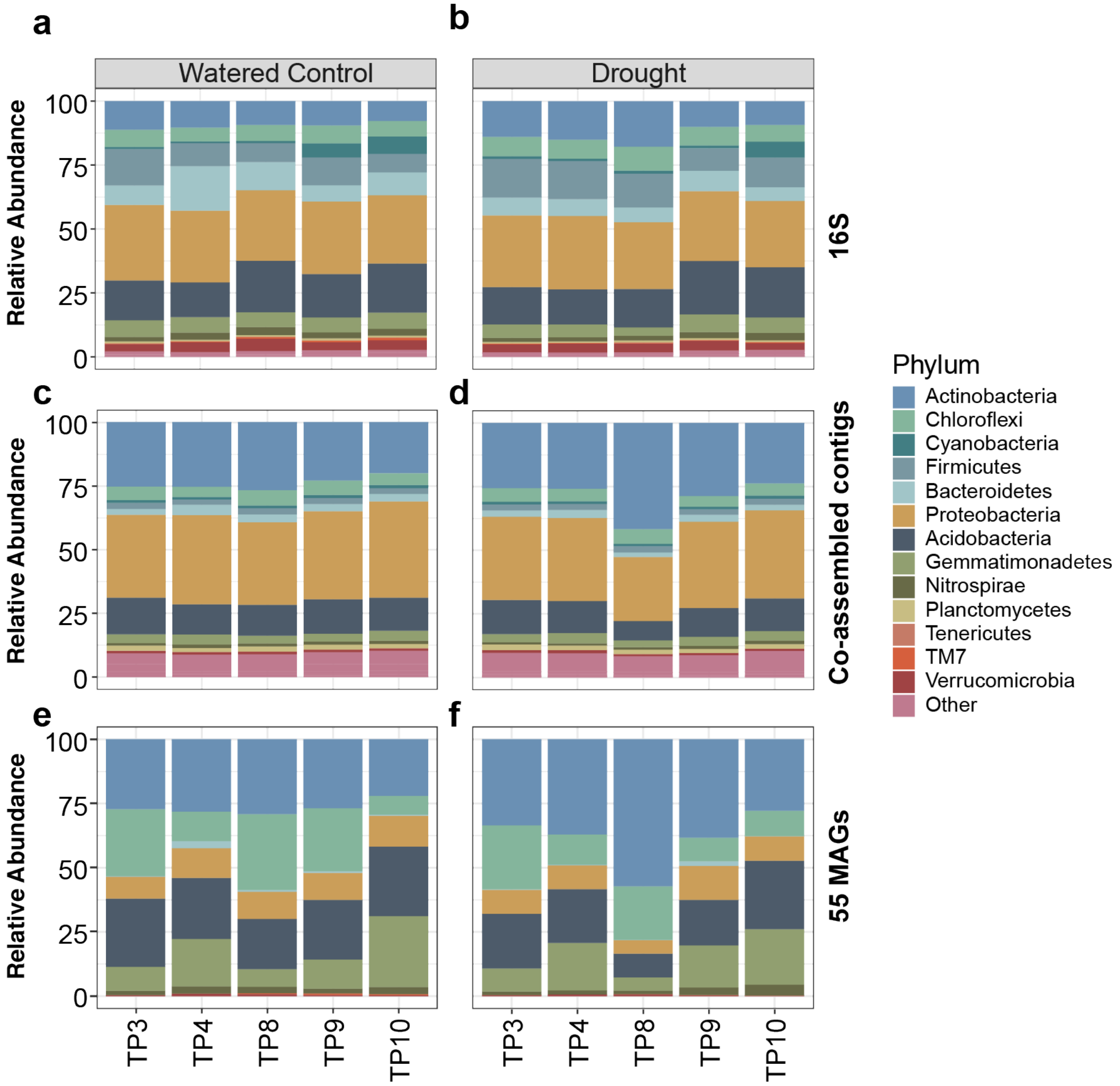

Supplementary Figure 4. Relative abundance profiles of microbial taxa in the bulk soil under drought. Percent relative abundance (y-axis) in the soil of the top 13 most abundant phyla for watered controls (**a**, **c**, and **e**) and drought treatments (**b**, **d** and **f**) treatments as measured by 16S rRNA gene amplicon sequencing (**a** and **b**), co-assembled contigs from shotgun metagenomic datasets (**c** and **d**), and all reads mapping to the 55 metagenome-assembled genomes (MAGs) (**e** and **f**). All five time points (TPs) (TP3-TP10, shown on the x-axis) were selected based on availability of data across all three data analysis types. All reads that mapped to other phyla or which were not classifiable at the phylum level are grouped into a fourteenth category, entitled “Other”.

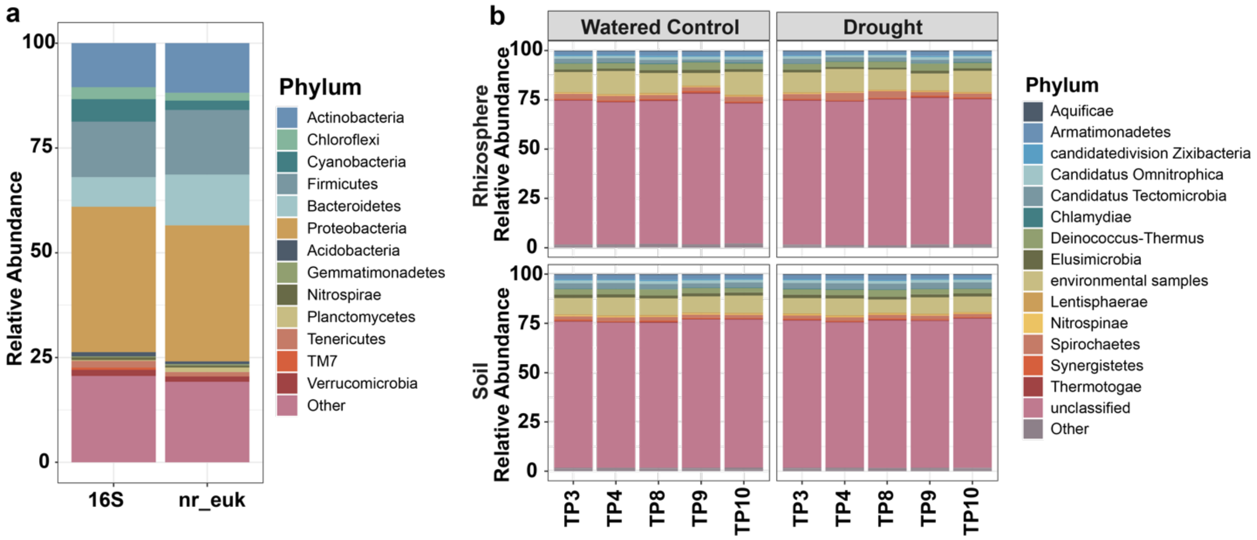

Supplementary Figure 5. Relative abundance plots across shotgun metagenomic datasets. a, Barplots of the relative abundance (representation) in the two databases used for taxonomic assignment of 16S (left bar) and shotgun (“nr_euk”, right bar) for the top 13 most abundant phyla observed within our 16S datasets (as shown in Fig 1). **b**, Barplots of relative abundance of the fifteen most abundant phyla present with shotgun reads mapping to “Other” in Figure 1. These correspond to the 14th through 27th most abundant phyla in the shotgun dataset. The majority of reads remain unclassified (shown in pink), while a small fraction (<1%) are classified within other less abundant phyla. Abundance within the rhizosphere and soils are indicated within the top and bottom row, respectively.

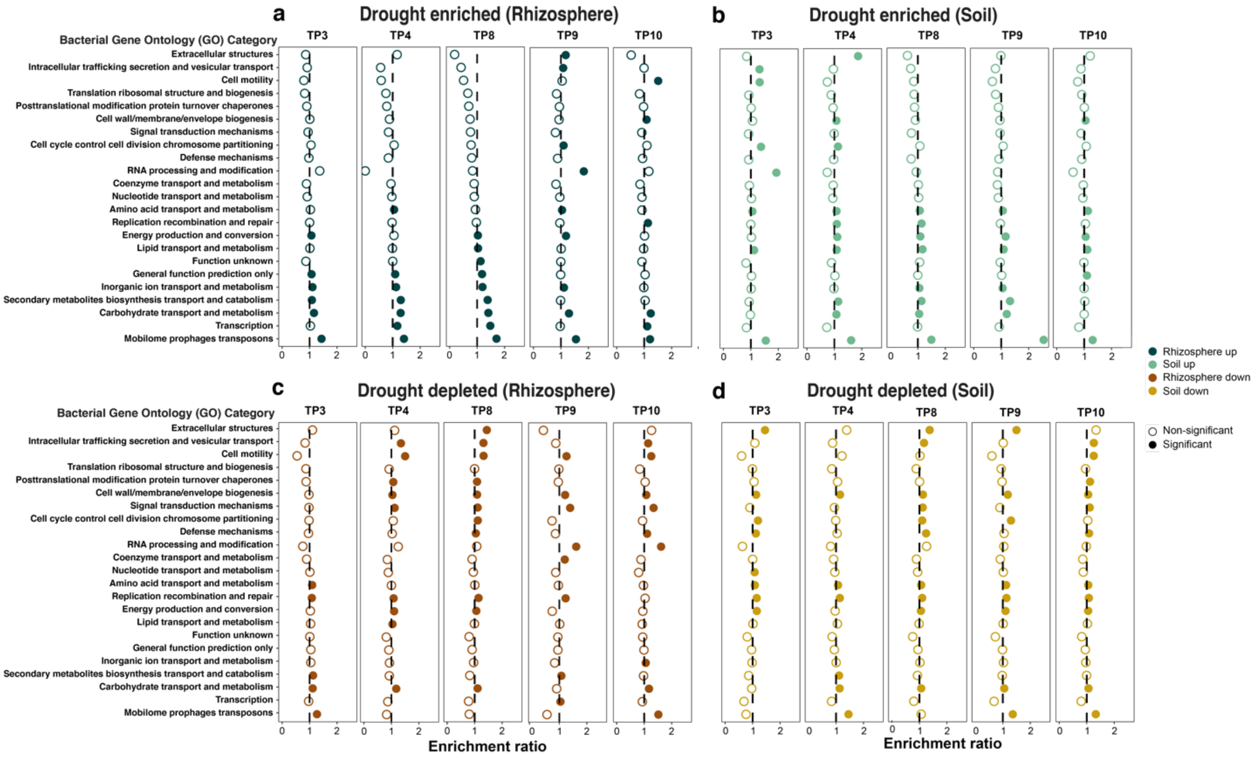

Supplementary Figure 6. Analysis of functional capacity in co-assembled rhizosphere and soil contigs under drought at TP3, TP4, TP8, TP9 and TP10

Bacterial Gene Ontology (GO) enrichment analysis for all genes derived from co-assembled contigs for the rhizospheres (**a, c**) and soils (**b, d**) across five time points (TP3, TP4, TP8, TP9, TP10). The values on the x axis indicate the fold enrichment ratio of the relative percentages of genes that are up-regulated (**a, b**) or down-regulated (**c, d**) under drought in each category relative to the total relative percentage of genes in the corresponding category within the entire dataset. Categories for which there were fewer than five differentially expressed genes were omitted. Solid circles indicate the enrichment was significant (p <= 0.05) in a one-sided hypergeometric test.

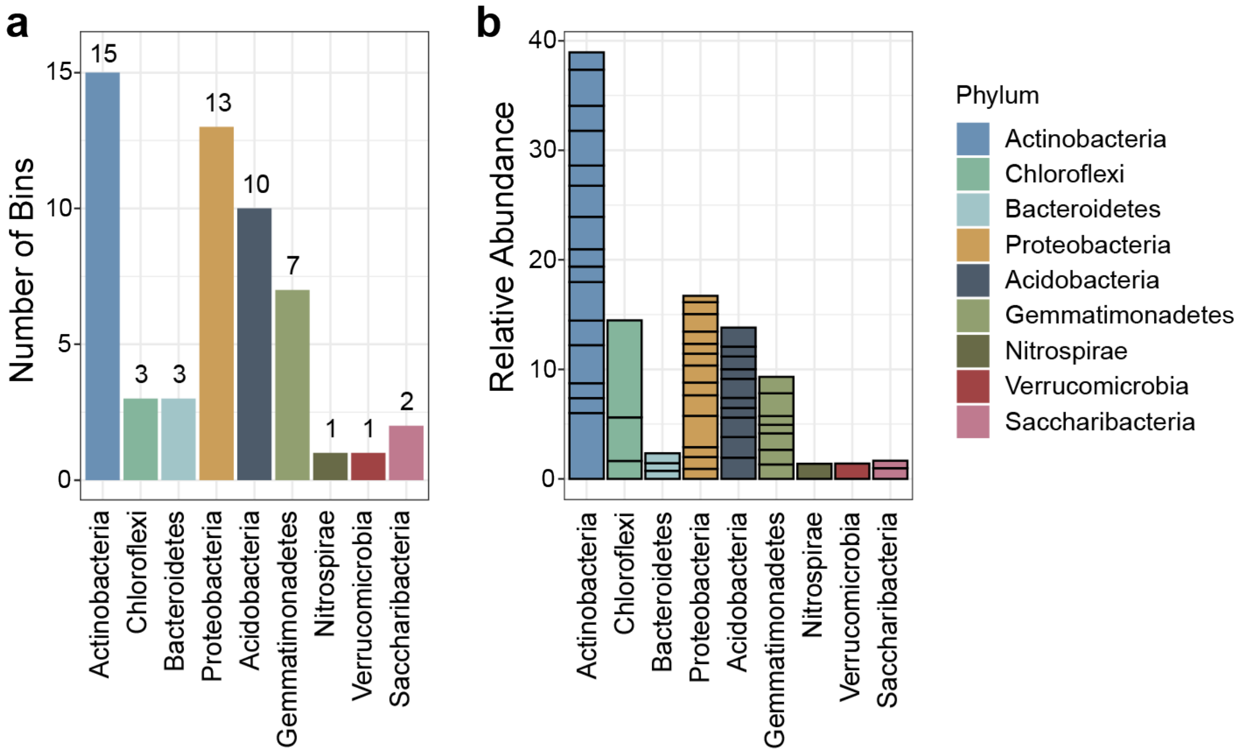

Supplementary Figure 7. Taxonomic diversity of the 55 metagenome-assembled genomes (MAGs). a, Barplot of the total number of bins (MAGs) belonging to each of the nine phylum represented by the genome-resolved metagenomic dataset. **b**, Shotgun metagenomic reads across all 52 samples were mapped to the 55 bins (MAGs) in our dataset. The stacked barplot displays the relative percentage of total reads that mapped to each bin, with bins belonging to the same phyla stacked to help visualize total per phyla mapping. The cumulative relative abundance summed across all bins is equal to 100 percent.

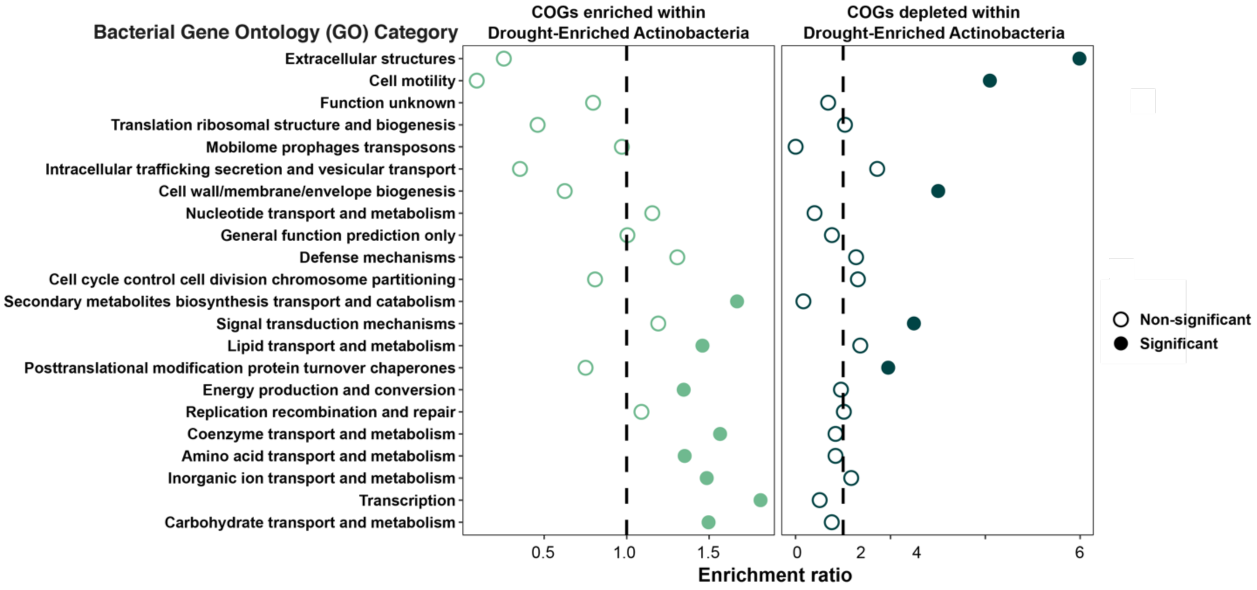

Supplementary Figure 8. Analysis of functional capacity of Drought-Enriched Actinobacterial metagenome-assembled genomes (MAGs) at TP8.

Bacterial Gene Ontology (GO) enrichment analysis for all COGs showing enrichment in enriched Actinobacterial MAGs as compared with all non-enriched bacterial MAGs under drought for rhizospheres at TP8. The values on the x axis indicate the fold enrichment ratio of the relative percentages of COGs up-regulated under drought in each category relative to the total relative percentage of COGs in the corresponding category within the entire dataset. The solid circles indicate categories for which the enrichment had a p value of < 0.05 in a one-sided hypergeometric test.

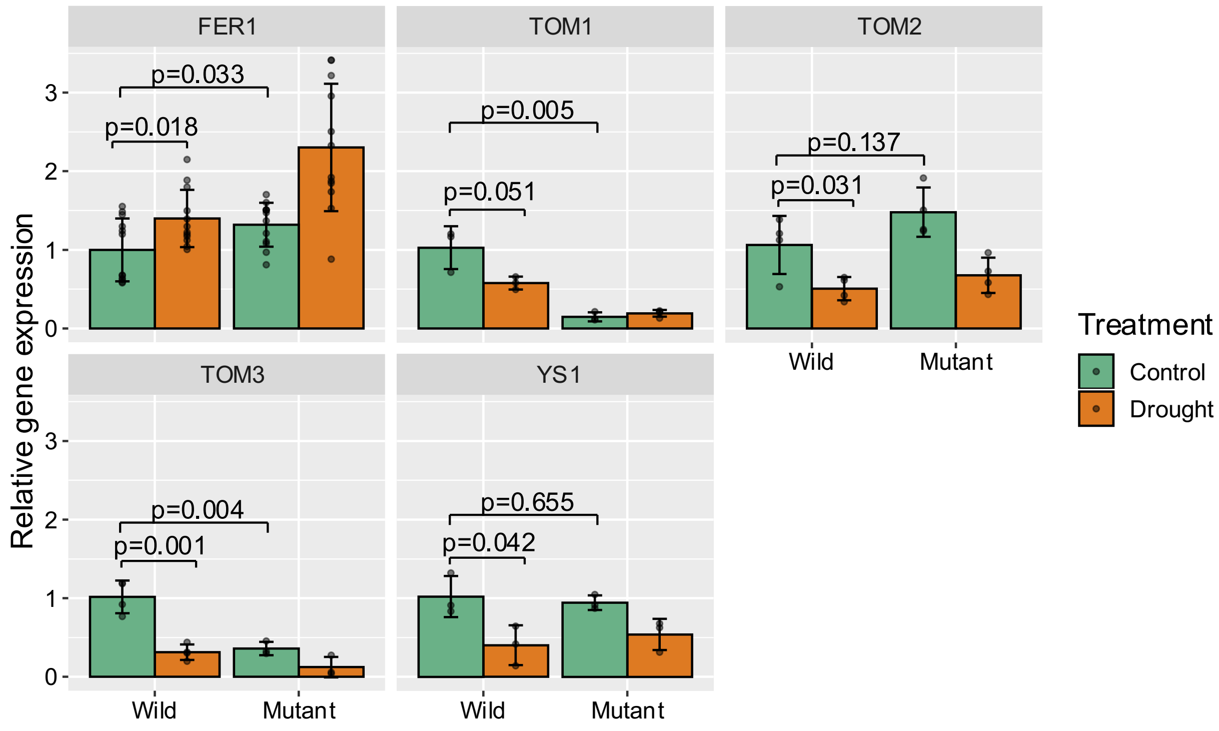

Supplementary Figure 9. qPCR with gene-specific primers in wild type maize and *tom1* mutant. Bar plots of the relative gene expression as measured by *qRT-PCR* for five genes (Ferritin1 (FER1), TOM1, TOM2, TOM3 and YS1) within WT and *tom1* mutant plants under both control and drought conditions (values are means ± SD (n = 4) from four independent biological replicates per treatment group, genotype and primer pair combination). *qPCR* was performed using gene specific primers and normalized to the plant housekeeping gene GAP2H. The y-axis represents the relative abundance of each gene, normalized to the abundance observed in wild type under control conditions. The x-axis for each plot is divided by genotype, and then by treatment. Differences between treatments and genotype were calculated with a two-sided paired t-test, and p values are indicated above pairs of bars for the specific comparisons discussed in the text.

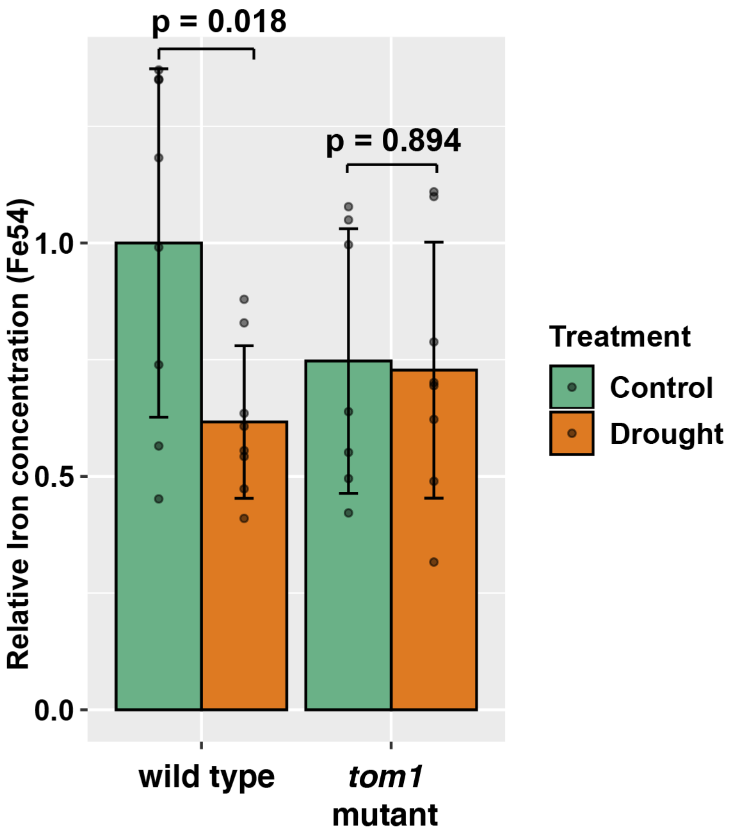

Supplementary Figure 10. Root iron concentration in *tom1* mutant and wild type maize under control and drought conditions. Barplot of absolute iron concentration as measured by ICP-MS (Fe54) within root tissue of wild type (left) and tom1 mutant (right) plants under both drought (orange) and control (green) conditions (values are means ± SD (n = 8) from eight independent biological replicates per treatment group, genotype and primer pair combination). The y-axis values are normalized to the mean value of iron levels observed for WT plants under control conditions (leftmost bar). Differences between treatments were calculated with a two-sided paired t-test.

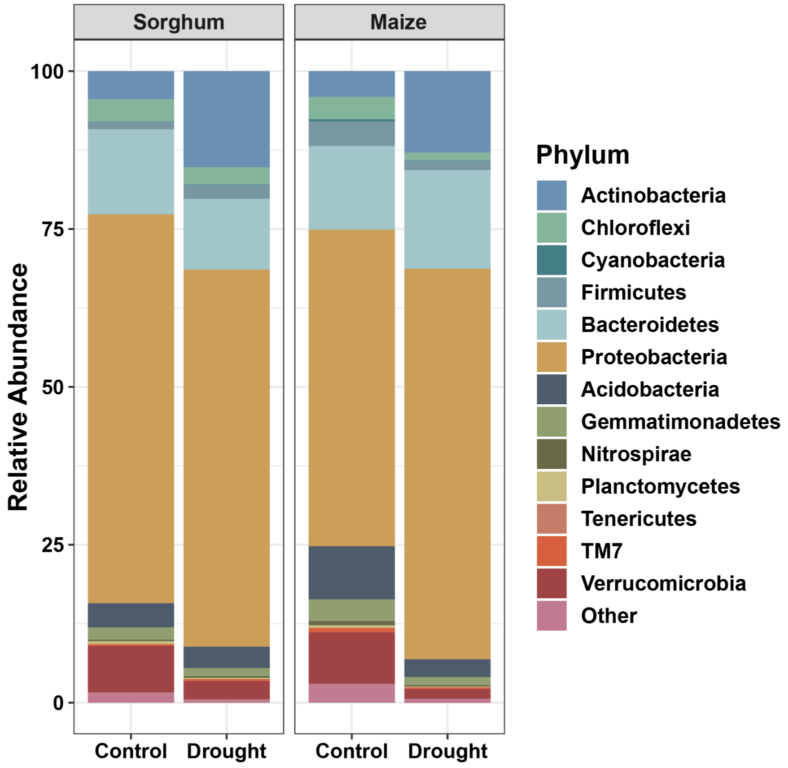

Supplementary Figure 11. Sorghum and maize rhizospheres share similar compositional responses to drought stress. Wild type sorghum and maize plants were grown in pots under greenhouse conditions in field soil as described for the *tom1* mutant experiment (see Methods) for four weeks, after which rhizosphere samples were collected. Percent relative abundance (y-axis) in the rhizosphere of the top 13 most abundant phyla for sorghum (left panel) and maize (right panel) under water and drought treatments as measured by 16S rRNA gene amplicon sequencing. Sorghum and maize plants were grown under greenhouse conditions in field soil as described in the Methods and drought treatment was implemented for two and a half weeks prior, after which rhizosphere samples were collected. All reads that mapped to other phyla or which were not classifiable at the phylum level are grouped into a fourteenth category, entitled “Other”.

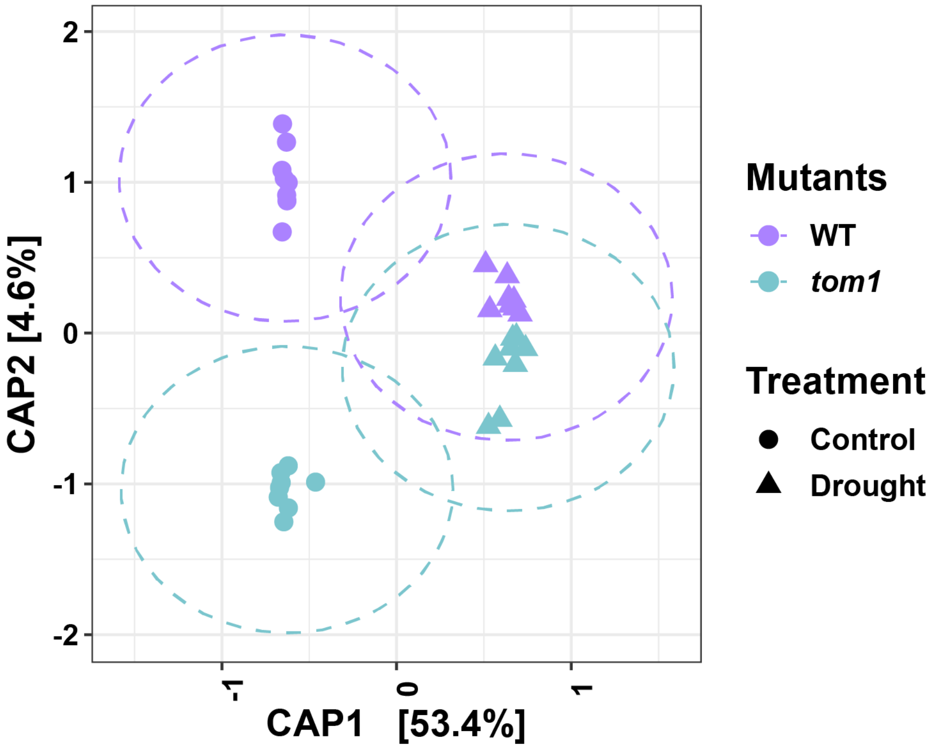

Supplementary Figure 12: TOM1 deficiency in plants shapes the composition of the root microbial community. Constrained ordination (CAPs) of rhizosphere microbiome composition showing the effect of plant genotype (Wild type or *tom1* mutant) under control and drought conditions. Ellipses show the parametric smallest area around the mean that contains 95% of the probability mass for each genotype. The bracketed numbers next to each CAP axis Label indicate the percent variation explained by that axis: 53.4% for treatment (CAP1), and 4.6% for genotype (CAP2).

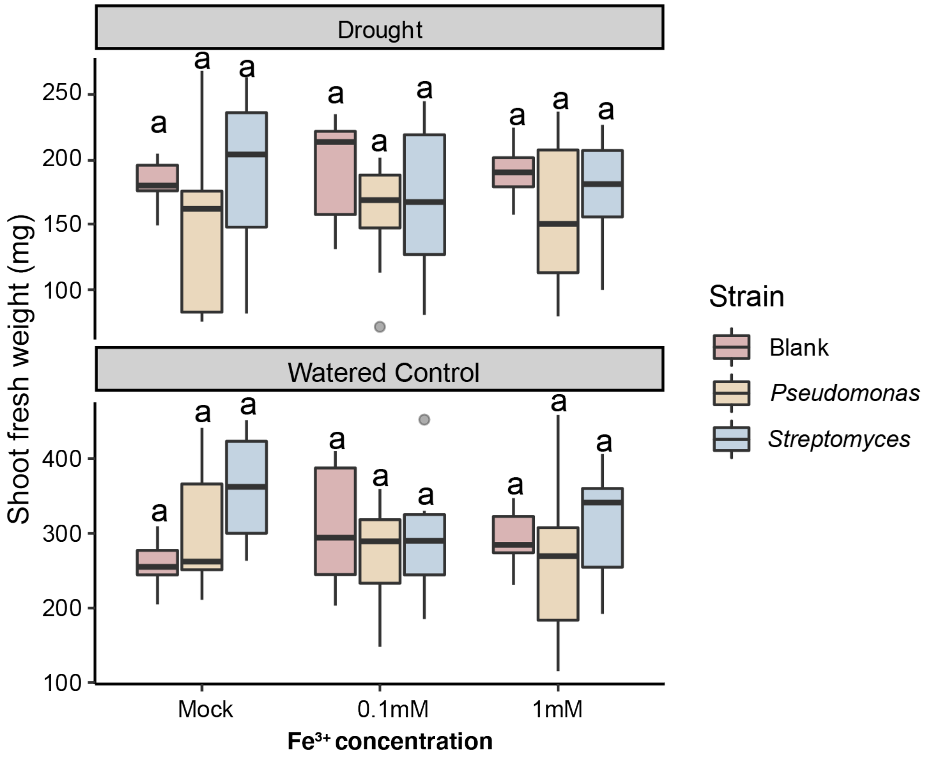

Supplementary Figure 13. Measurement of shoot fresh weight phenotypes upon application of two levels of Fe^{3+} and a mock control. Boxplots showing mean shoot fresh weight from the same experiment as shown in Figure 7, across three inoculation types (Blank, Streptomyces and Pseudomonas) and three iron supplementation levels (mock, 0.1mM and 1mM) (values are means ± SD (n = 7) from seven independent biological replicates per treatment group, inoculation, and iron supplementation combination). Plots are shown for both drought stress (top panel) and watered control (bottom panel). Box bounds indicate one quartile above and below mean, while whiskers indicate one standard deviation above the mean. No significant differences were observed in shoot fresh weight as a result of inoculation type following one-sided Tukey's multi comparison test with one-sided ANOVA. Tukey's groups, as specified by the letters (a) above each bar, indicate groups for which no significant difference (p value < 0.05) was present between members of the group.

Supplementary Tables

Supplementary Table 1. Primer sequences used in this study for qRT-PCR. The Gene name (as described in the manuscript), Gene ID, Primer name and Primer sequence are shown. Primers for Ferritin 1 gene expression were designed in this study, whereas other primers were based on prior published work (see References column).

Gene name	Gene ID or Accession Number	Primer name	Primer sequence	References
Ferritin1	Sobic.008G004800	Fer1-1L	CCTCTTCGCCTACTTCGACC	This study
Ferritin1	Sobic.008G004800	Fer1-2L	AAGTTCGTCGATGACTGCGA	This study
Ferritin1	Sobic.008G004800	Fer1-1R	AGAGCCAGCTCCATAGCGTA	This study
Ferritin1	Sobic.008G004800	Fer1-2R	CCAGCTCCATAGCGTACAGA	This study
Ferritin1	Sobic.008G004800	Fer1-3L	CTCAACGAGCAGATCAACGTG	This study
Ferritin1	Sobic.008G004800	Fer1-3R	AATCGATTGGAGCCTCACCC	This study
GAPDH	X07156	GAPDH-L	CCATCACTGCCACACAGAAAAC	105
GAPDH	X07156	GAPDH-R	AGGAACACGGAAGGACATACCAG	105
TOM1	GRMAM2G063306	TOM1L	AAGTGTAAATTCATGCCGTG	35
TOM1	GRMAM2G063306	TOM1R	GATCCCCTGGAAAGAAGGCA	35
TOM2	GRMZM5G877788	TOM2L	GTTTCGTCGGCGCTATCCAT	35
TOM2	GRMZM5G877788	TOM2R	AAGAACGCGGCATGCTGGCG	35
TOM3	GRMZM2G141081	TOM3L	GTGCTTTCAGTCACAGGCGT	35
TOM3	GRMZM2G141081	TOM3R	ATAGGTCCAAGGATTTTGTT	35
YS1	GRMZM2G156599	YS1L	CACTCATCAGCAAGCAGAAA	35
YS1	GRMZM2G156599	YS1R	TGCAGACTCTTAACAGTGAC	35
