# Supplementary material for: Genome-resolved metagenomics reveals role of iron metabolism in drought-induced rhizosphere microbiome dynamics
Source: Nat Commun. 2021 May 28;12:3209. doi: 10.1038/s41467-021-23553-7 (PMC8163885; doi:10.1038/s41467-021-23553-7)
Supplement: Supplementary file 6 — Reporting Summary [file 41467_2021_23553_MOESM6_ESM.pdf]

nature research

Corresponding author(s): Coleman-Derr
Last updated by author(s): Apr 10, 2021

Reporting Summary

Nature Research wishes to improve the reproducibility of the work that we publish. This form provides structure for consistency and transparency in reporting. For further information on Nature Research policies, see our Editorial Policies and the Editorial Policy Checklist.

Statistics

For all statistical analyses, confirm that the following items are present in the figure legend, table legend, main text, or Methods section.

n/a	Confirmed	
☐	☒	The exact sample size (n) for each experimental group/condition, given as a discrete number and unit of measurement
☐	☒	A statement on whether measurements were taken from distinct samples or whether the same sample was measured repeatedly
☐	☒	The statistical test(s) used AND whether they are one- or two-sided<br>*Only common tests should be described solely by name; describe more complex techniques in the Methods section.*
☐	☒	A description of all covariates tested
☐	☒	A description of any assumptions or corrections, such as tests of normality and adjustment for multiple comparisons
☐	☒	A full description of the statistical parameters including central tendency (e.g. means) or other basic estimates (e.g. regression coefficient) AND variation (e.g. standard deviation) or associated estimates of uncertainty (e.g. confidence intervals)
☐	☒	For null hypothesis testing, the test statistic (e.g. F, t, r) with confidence intervals, effect sizes, degrees of freedom and P value noted<br>*Give P values as exact values whenever suitable.*
☒	☐	For Bayesian analysis, information on the choice of priors and Markov chain Monte Carlo settings
☒	☐	For hierarchical and complex designs, identification of the appropriate level for tests and full reporting of outcomes
☒	☐	Estimates of effect sizes (e.g. Cohen's d, Pearson's r), indicating how they were calculated

Our web collection on statistics for biologists contains articles on many of the points above.

Software and code

Policy information about availability of computer code

Data collection	No code was used to collect data
Data analysis	The analysis code that supports the findings of this study is available at GitHub https://github.com/LingXu2018/EPICON_IRON.<br>The following software packages were used as part of this study.<br><br>Kallisto v0.42.4<br>DESeq2 v0.4.3<br>megahit v1.1.2<br>Bowtie2 v2.3.4<br>MetaBAT v2.12.1<br>DAS Tool v1.1.1<br>CheckM v1.0.11<br>GTDB-Tk v1.3.0<br>FastTree v2.1<br>FastQC v0.11.9<br>cutadapt v1.16<br>Kaiju v1.6.3<br>Prokka v1.13<br>dRep v2.0.5<br>edgeR v3.24.3<br>CONCOCT v1.1.0<br>MaxBin v2.0

itagger v2.0.0
RDP v2.9.0
HISAT v2.1.0
BBDuk v38.88
featureCounts 1.22.2
pheatmap 1.12.0

For manuscripts utilizing custom algorithms or software that are central to the research but not yet described in published literature, software must be made available to editors and reviewers. We strongly encourage code deposition in a community repository (e.g. GitHub). See the Nature Research guidelines for submitting code & software for further information.

Data

Policy information about availability of data

All manuscripts must include a data availability statement. This statement should provide the following information, where applicable:

- Accession codes, unique identifiers, or web links for publicly available datasets
- A list of figures that have associated raw data
- A description of any restrictions on data availability

The sequences reported in this paper have been dposited in the National Center for Biotechnology inofrmation Sequence Read Archive, www.ncbi.nlm.nih.gov (accession number PRJNA435634).

Field-specific reporting

Please select the one below that is the best fit for your research. If you are not sure, read the appropriate sections before making your selection.

☒ Life sciences ☐ Behavioural & social sciences ☐ Ecological, evolutionary & environmental sciences

For a reference copy of the document with all sections, see nature.com/documents/nr-reporting-summary-flat.pdf

Life sciences study design

All studies must disclose on these points even when the disclosure is negative.

Sample size	For experiments in this study involving rhizosphere and soil samples collected for shotgun analyses, transcriptomics and 16S rRNA analyses, an n=3 was chosen per time point and treatment, with a total of 52 samples for the dataset, as in our prior publication for microbiome analysis and transcriptomics (see citations in the manuscript for Xu et al 2018, PNAS and Varoquaox et al 2019, PNAS). For experiments in this study involving qRT-PCR of maize tom1 plants under drought stress, an n=3 was chosen, as in our prior publications (see citations in the manuscript for Xu et al 2018, PNAS).
Data exclusions	No data were excluded beyond sequencing reads with low quality scores.
Replication	Experiments were not replicated more than once due to the cost of materials, library preparation and sequencing, unless stated otherwise in the text and stated hereafter. Triplicate PCR reactions were performed for 16S library preparation and pooled to control for variation in PCR quality and stochastic effects due to variation in template quality and quantity.
Randomization	Sample collection for the field experiment used for shotgun metagenomics were harvested from a randomized block design and two different types of irrigation (drought stress and watered controls) with three replicate blocks per treatment. Blocks were distrubuted randomly within the field, and samples were collected at random from within each treatment and replicate block at each time point. For qRT-PCR experiments, replicate boxes were positioned in random locations within the growth chamber and were repositioned on a daily bases to control for variation in lighting and temperature within the chamber.
Blinding	Blinding for phenotypic data was not possible as experimental conditions were evident from the samples collected (drought and control tissues differ in quality and quantity). For sequence based analyes of shotgun, 16S, transcriptomics datasets, blinding was not relevant as quantifications were performed using computational pipelines applied equally to all conditions and replicates and it is not possible that researchers could have influenced sequencing results in a systematic manner.

Reporting for specific materials, systems and methods

We require information from authors about some types of materials, experimental systems and methods used in many studies. Here, indicate whether each material, system or method listed is relevant to your study. If you are not sure if a list item applies to your research, read the appropriate section before selecting a response.

Materials & experimental systems

n/a	Involved in the study
☒	☐ Antibodies
☒	☐ Eukaryotic cell lines
☒	☐ Palaeontology and archaeology
☒	☐ Animals and other organisms
☒	☐ Human research participants
☒	☐ Clinical data
☒	☐ Dual use research of concern

Methods

n/a	Involved in the study
☒	☐ ChIP-seq
☒	☐ Flow cytometry
☒	☐ MRI-based neuroimaging
